# Inference of single-cell network using mutual information for scRNA-seq data analysis

**DOI:** 10.1186/s12859-024-05895-3

**Published:** 2024-09-05

**Authors:** Lan-Yun Chang, Ting-Yi Hao, Wei-Jie Wang, Chun-Yu Lin

**Affiliations:** 1 Institute of Bioinformatics and Systems Biology, National Yang Ming Chiao Tung University, Hsinchu, 300 Taiwan; 2 Department of Biological Science and Technology, National Yang Ming Chiao Tung University, Hsinchu, 300 Taiwan; 3 Institute of Data Science and Engineering, National Yang Ming Chiao Tung University, Hsinchu, 300 Taiwan; 4 Center for Intelligent Drug Systems and Smart Bio-Devices, National Yang Ming Chiao Tung University, Hsinchu, 300 Taiwan; 5 Cancer and Immunology Research Center, National Yang Ming Chiao Tung University, Taipei, 112 Taiwan; 6 School of Dentistry, Kaohsiung Medical University, Kaohsiung, 807 Taiwan

**Keywords:** Single-cell analysis, Network inference, Mutual information, Gene expression, Single-cell network, Single-cell clustering

## Abstract

**Background:**

With the advance in single-cell RNA sequencing (scRNA-seq) technology, deriving inherent biological system information from expression profiles at a single-cell resolution has become possible. It has been known that network modeling by estimating the associations between genes could better reveal dynamic changes in biological systems. However, accurately constructing a single-cell network (SCN) to capture the network architecture of each cell and further explore cell-to-cell heterogeneity remains challenging.

**Results:**

We introduce SINUM, a method for constructing the SIngle-cell Network Using Mutual information, which estimates mutual information between any two genes from scRNA-seq data to determine whether they are dependent or independent in a specific cell. Experiments on various scRNA-seq datasets with different cell numbers based on eight performance indexes (e.g., adjusted rand index and F-measure index) validated the accuracy and robustness of SINUM in cell type identification, superior to the state-of-the-art SCN inference method. Additionally, the SINUM SCNs exhibit high overlap with the human interactome and possess the scale-free property.

**Conclusions:**

SINUM presents a view of biological systems at the network level to detect cell-type marker genes/gene pairs and investigate time-dependent changes in gene associations during embryo development. Codes for SINUM are freely available at https://github.com/SysMednet/SINUM.

**Supplementary Information:**

The online version contains supplementary material available at 10.1186/s12859-024-05895-3.

## Background

Hundreds of cell types manifest different morphologies, behaviors, and functions, although every cell in the human body contains nearly identical genomic information [1]. To unravel the homogeneity or heterogeneity among a population of cells, coordinated gene expression in cells plays a crucial role in revealing biological function and cellular responses, especially when disease occurs [2]. Recently, the advance in single-cell RNA sequencing (scRNA-Seq) technology enables mass production of single-cell transcriptomic data and offers an attractive alternative to explore genetic and functional heterogeneity with cellular resolution via expression profiling studies; for example, the discovery of unidentified cell types [3], human embryonic development [4], and intra- and inter-tumoral heterogeneity [5, 6]. It has been known that it is difficult to understand the gene functions and the effects of disease-associated variants at the individual gene level due to the complex and dynamic associations among these genes, which constitute a complicated network [7, 8]. However, most studies still rely on differential expression analysis on scRNA-seq data [9–11] but overlook the biological networks, which play a key role in the current development of biomarkers and therapeutic targets in disease [12–14].

In support of this pursuit, previous works have proposed several methods to estimate networks using gene expression data, such as on the basis of Boolean models [15], correlation models [16, 17], and information models [18, 19] Yet, recent research has indicated that Boolean model-based methods have limited scalability when constructing a genome-scale network; additionally, a large part of the variation for correlation-based approaches may originate from a variety of technical factors, which can easily produce confounding effects in correlation inference [20]. Unlike the Pearson correlation coefficient, which evaluates linear correlations between the measured variables, mutual information (MI) as an information model is able to capture non-linear and non-monotonic relationships for representing the dynamic relationships between pairs or groups of genes more accurately [18, 21, 22]. MI is calculated by estimating pairwise joint probability distributions and typically requires density estimation or data discretization, and the quality of these estimates depends on sample size [23, 24]. Thus, scRNA-seq data is suitable for an MI measure because the sample size is sufficiently large. One MI-based network inference approach, called partial information decomposition and context (PIDC), has been designed for single-cell transcriptomic data analysis [18]. However, PIDC requires scRNA-seq data from a group of cells to build an aggregate network (i.e., one network for a group of cells), and it may be difficult to fully employ the advantages of single-cell technology to explore cell-to-cell heterogeneity. Therefore, another information-based method, cell-specific network (CSN), has recently been proposed to construct the single-cell network (SCN) [19]. The CSN method estimates the gene–gene association by statistical independence of two genes based on the joint density function, which is equal to the product of two marginal density functions when two genes are independent. However, CSN may underestimate some gene–gene associations that only occur in a small subgroup of cells because the theoretical model assumes an association if two genes are dependent in a certain amount of cells. For example, the SCNs inferred by the CSN method from seven scRNA-seq datasets display a low overlap (~ 3%) with the two human protein‒protein interaction (PPI) networks (Additional file 1: Table 1), collected from the Search Tool for the Retrieval of Interacting Genes/Proteins (STRING) database [25] and assembled from 21 public databases by Gysi et al*.* [26]. Thus, accurately extracting the network architecture of each cell from such diverse scRNA-seq datasets is still an urgent and unmet need.

To address these issues, we propose a SIngle-cell Network Using Mutual information (SINUM) method to infer SCNs from the scRNA-seq data (i.e., one network for one cell). SINUM integrates a measure of MI with the hypotheses of various dependent relations used in CSN to determine whether any given two genes are dependent (an edge) or independent (no edge) in a specific cell and further builds the SCN (undirected network). Moreover, SINUM SCNs can transform into the network degree matrix (DM) by counting and normalizing the number of edges connected to every gene in each SCN. Specifically, DM has the same dimension as the original gene expression matrix (GEM) (i.e., *m* genes × *n* cells) and can be directly used in most of the subsequent scRNA-seq analyses. Using the seven scRNA-seq datasets, we validated the effectiveness of our SINUM in distinguishing cell types, which outperforms the CSN method and direct use of the original GEM. Compared to CSN SCNs, the SINUM SCNs are more likely to fit the scale-free characteristics and display a higher overlap with the two human PPI networks. Additionally, our SINUM can be applied to identify cell-type marker genes and gene pairs, which have a differential network degree between a specific cell type and the others but no differential gene expression, and study time-dependent changes in gene associations during embryo development. We believe that SINUM offers a route of access for identifying gene–gene associations to construct networks at a single-cell resolution, provides a new opportunity to facilitate the identification of cell types and biomarkers, and presents a view of biological systems at the network level to compensate for the current analyses of scRNA-seq data.

## Materials and methods

### Construction of single-cell network using mutual information (*SINUM*)

To determine the gene‒gene association of genes *X* and *Y* in a specific cell, we applied MI to quantify the mutual dependency between their expression values, $G_{X}$ and $G_{Y}$. MI score for ${ }G_{X}$ and $G_{Y}$ is defined as

1 $$I\left( {G_{X} ;G_{Y} } \right) = H\left( {G_{X} } \right) + H\left( {G_{Y} } \right) - H\left( {G_{X} ,G_{Y} } \right)$$

where $H\left( {G_{X} } \right)$, $H\left( {G_{Y} } \right)$, and $H\left( {G_{X} ,G_{Y} } \right)$ are the entropy of $G_{X}$, entropy of $G_{Y}$, and joint entropy of $G_{X}$ and $G_{Y}$, respectively. In information theory, MI as a similarity metric provides symmetric, i.e., $I\left( {G_{X} ;G_{Y} } \right) = I\left( {G_{Y} ;G_{X} } \right)$, and non-negative, i.e., $I\left( {G_{X} ;G_{Y} } \right) \ge 0$, measurement to determine the statistical dependency between two random variables (i.e., two genes in this work) [27]. In other words, MI quantifies how much a random variable reveals the other and could be interpreted as reducing uncertainty about one when given the knowledge of the other. When assessing the dependency, the higher the MI measure for a given gene pair, the stronger the coordinated expression between these two genes [18].

**Fig. 1:**
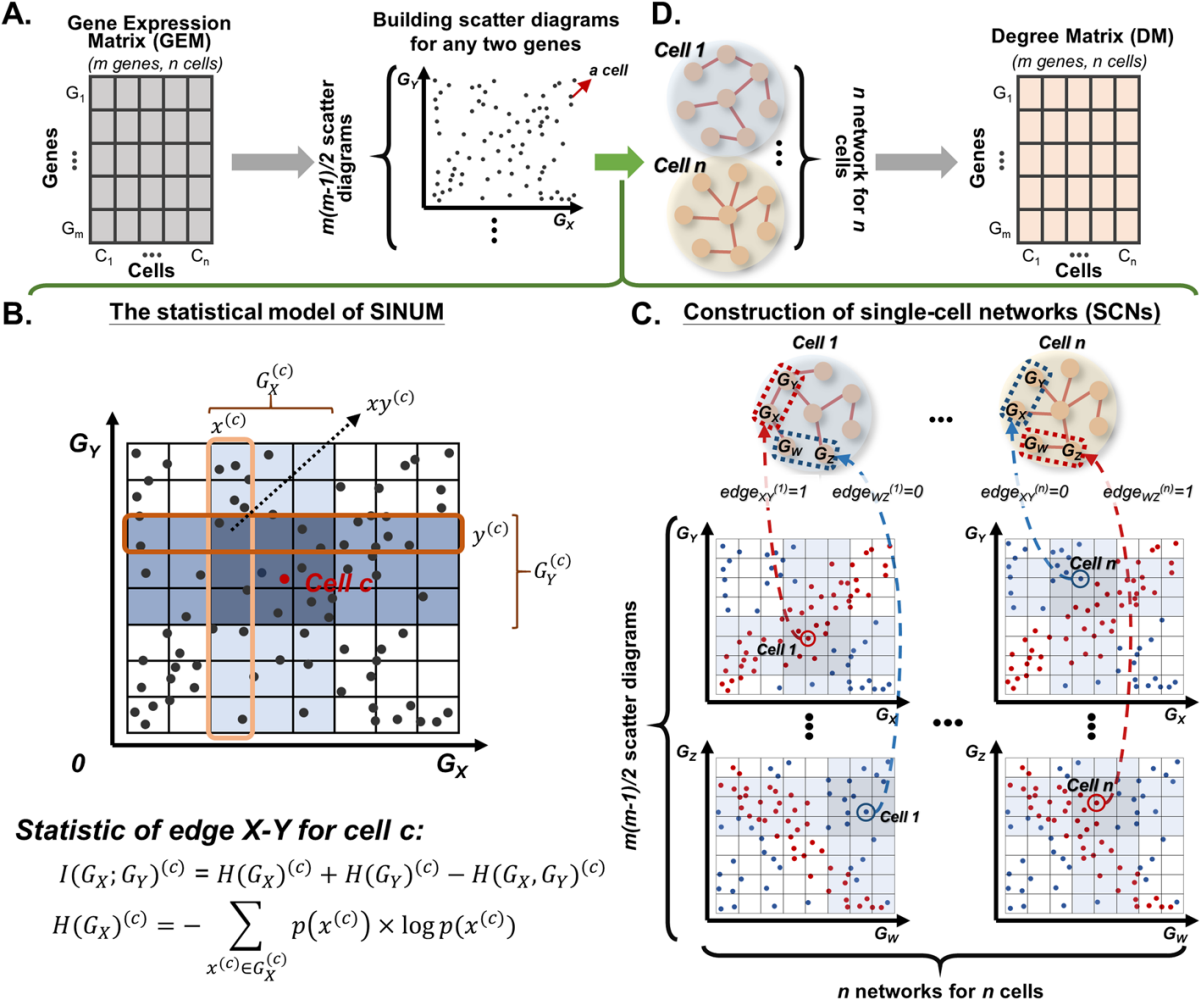
Overview of SINUM (SIngle-cell Network Using Mutual information) method. **A** Generation of scatter diagrams for every two genes from the gene expression matrix (GEM), where the *x*- and *y*-axes are the expression values of every two genes within the *n* cells. Each point denotes a cell. **B** The statistical model of SINUM for estimating the association between genes *X* and *Y*. First, each scatter diagram containing *n* cells was split into $\lfloor\sqrt n + \frac{1}{2}\rfloor$ grids. Next, SINUM produces two boxes $G_{X}^{\left( c \right)}$ (light blue) and $G_{Y}^{\left( c \right)}$ (medium blue) close to cell *c* to represent its neighborhoods of expression values for genes *X* and *Y*, respectively; thus, the intersection region can directly produce the third box $G_{XY}^{\left( c \right)}$ (dark blue). The entropies $H\left( {G_{X} } \right)^{\left( c \right)}$, $H\left( {G_{Y} } \right)^{\left( c \right)}$, and $H\left( {G_{X} ,G_{Y} } \right)^{\left( c \right)}$ of the boxes $G_{X}^{\left( c \right)}$, $G_{Y}^{\left( c \right)}$, and $G_{XY}^{\left( c \right)}$, respectively, were then evaluated for calculating mutual information, $I\left( {G_{X} ;G_{Y} } \right)^{\left( c \right)}$. The mutual information is used to evaluate whether any given two genes *X* and *Y*, are a dependent or independent gene pair in cell *c* among all cells. If the value of $I\left( {G_{X} ;G_{Y} } \right)^{\left( c \right)}$ is larger than the threshold, it suggests that genes *X* and *Y* are dependent on each other in cell *c* and will be represented by an edge in a network. **C** Construction of *n* single-cell networks (SCNs) for *n* cells. For *m* genes, a total of $m\left( {m - 1} \right)/2$ scatter diagrams were generated to measure all possible associations between two genes. In each SCN, the red solid line represents that there’s an edge between two genes for a specific cell inferred by our SINUM method; otherwise, there’s no edge. **D** Generation of degree matrix (DM) by counting the number of edges connected to every gene in each SCN. Note that the size of DM is the same as GEM with *m* rows and *n* columns

To estimate MI for determining an edge between two genes on scRNA-seq data, we first generated a scatter diagram for every two genes from the GEM, i.e., *m* genes lead to $m\left( {m - 1} \right)/2$ scatter diagrams, where each data point denotes a cell; *x*- and *y*-axes represent the expression values of these two genes in the *n* cells (Fig. 1A). The scatter diagram was further equally split into $GR$ grids according to the total number of cells (here is *n*) and the distribution of cells; in other words, the minimum and maximum expression values of each two genes in the *n* cells were used to determine the overall boundaries and adjust the grid size. Thus, $GR$ is the number of grids and given as

2 $$GR = \lfloor\sqrt n + \frac{1}{2}\rfloor$$

If the cells are uniformly randomly distributed in the scatter diagram for two genes, we can expect the number of cells in each grid to be nearly equal; accordingly, the two genes are independent of each other. Then, we defined the tentative neighborhoods for the target cell *c* based on the given box size. The box size was used to determine the neighborhood size; for example, the box size of 0.2 means that there are 20% of *n* cells within the tentative neighborhood of cell *c*. To reduce the intrinsic fluctuation of gene expression values in scRNA-seq data, the tentative neighborhood was expanded outward to the closest grid boundaries and formed the final neighborhoods, $G_{X}^{\left( c \right)}$ (light blue) and $G_{Y}^{\left( c \right)}$(medium blue), for genes *X* and *Y*, respectively (Fig. 1B). Within the final neighborhoods $G_{X}^{\left( c \right)}$ and $G_{Y}^{\left( c \right)}$, several sub-regions $x^{\left( c \right)}$ and $y^{\left( c \right)}$, could be identified based on the corresponding grids, respectively; in other words, $x^{\left( c \right)}$ or $y^{\left( c \right)}$ is separately a column or row of grids belonging to $G_{X}^{\left( c \right)}$ or $G_{Y}^{\left( c \right)}$. The intersection of the two final neighborhoods $G_{X}^{\left( c \right)}$ and $G_{Y}^{\left( c \right)}$ can directly produce the third box, called $G_{XY}^{\left( c \right)}$ (dark blue), and is also divided into several sub-regions $xy^{\left( c \right)}$ (Fig. 1B).

In this study, we derived a local measurement from Eq. (1) to evaluate whether any given two genes *X* and *Y*, are dependent or independent in cell *c*. Thus, the MI score, $I\left( {G_{X} ;G_{Y} } \right)^{\left( c \right)}$, is given as $I\left( {G_{X} ;G_{Y} } \right)^{\left( c \right)} = H\left( {G_{X} } \right)^{\left( c \right)} + H\left( {G_{Y} } \right)^{\left( c \right)} - H\left( {G_{X} ,G_{Y} } \right)^{\left( c \right)}$. Then, the uncertainty and randomness of a random variable (i.e., a gene) in the probability distribution could be quantified by a measure of entropy. The entropy of $G_{X}$ in cell *c*, $H\left( {G_{X} } \right)^{\left( c \right)}$, is calculated as

3 $$H\left( {G_{X} } \right)^{\left( c \right)} = - \mathop \sum \limits_{{x^{\left( c \right)} \in G_{X}^{\left( c \right)} }} p\left( {x^{\left( c \right)} } \right) \times \log p\left( {x^{\left( c \right)} } \right)$$

where $p\left( {x^{\left( c \right)} } \right)$ is the probability for $x^{\left( c \right)}$. Let $n_{x}^{\left( c \right)}$, $n_{y}^{\left( c \right)}$, and $n_{xy}^{\left( c \right)}$ denote the number of cells inside $x^{\left( c \right)}$, $y^{\left( c \right)}$ and $xy^{\left( c \right)}$, respectively, and the probability $p\left( {x^{\left( c \right)} } \right)$ can be substituted by the frequency numerically:

4 $$p\left( {x^{\left( c \right)} } \right) = \frac{{n_{x}^{\left( c \right)} }}{n}$$

$H\left( {G_{Y} } \right)^{\left( c \right)}$ and $H\left( {G_{X} ,G_{Y} } \right)^{\left( c \right)}$ were defined by following the same manners.

Finally, we transformed the MI score obtained from entropy for every gene pair to a *z* score, $z_{XY}^{\left( c \right)}$. $z_{XY}^{\left( c \right)}$ is defined as

5 $$z_{XY}^{\left( c \right)} = \frac{{I\left( {G_{X} ;G_{Y} } \right)^{\left( c \right)} - \mu_{XY} }}{{\sigma_{XY} }}$$

where $z_{XY}^{\left( c \right)}$ denotes the significant level of the MI score between genes *X* and *Y* in cell *c*; $\mu_{XY}$ and $\sigma_{XY}$ are the mean and standard deviation of MI scores, respectively, between genes *X* and *Y* across all cells. Here, the *z* score is used to determine the presence of an edge (association) between any given two genes in a single-cell network (SCN). There is an edge if its *z* score reaches above the threshold (Fig. 1C). For all gene pairs and cells, we could eventually construct *n* SCNs for *n* cells after repeating this procedure. Like the GEM, the DM was constructed by counting and normalizing the number of edges connected to each gene in each SCN (Additional file 1: Note S1); thus, DM has the same column and row numbers as the GEM (Fig. 1D).

### Network-based clustering with dimension-reduction for identifying cell types

Recently, the DM has been used to provide new insights from network science perspectives in applying the existing scRNA-seq technique, including dimensionality-reduction, clustering, and visualization [19]. Here, we first applied the principal component analysis (PCA) [28] to reduce the DM (or GEM) to 20 dimensions and further reduce it to two dimensions for visualization using the t-distributed stochastic neighbor embedding (t-SNE) [29]. PCA and t-SNE represent linear and non-linear methods of dimensionality reduction, respectively. Second, we implemented *k*-means and hierarchical algorithms to perform clustering analysis. The *k*-means algorithm clusters data by separating samples into *k* groups of equal variance via minimizing the sum of the distances between the centroid and all member objects of the group. This algorithm requires the number of clusters to be specified. On the other hand, the hierarchical clustering builds nested clusters by merging or splitting them successively. This hierarchy of clusters is represented as a dendrogram, in which the root is the unique cluster that gathers all the samples and the leaves are the clusters with only one sample. Finally, we evaluated the clustering performances of different methods mainly based on eight performance indexes, including adjusted rand index (ARI), F-measure index (FMI), adjusted mutual information (AMI), completeness scores (CPT), Fowlkes-Mallows scores (FMS), homogeneity scores (HMG), and normalized mutual information (NMI). Note that we set the same parameters when performing dimension-reduction, clustering, and performance evaluation for SINUM DMs, CSN DMs, and GEMs.

### Pre-processing of scRNA-seq data

The SCNs were constructed by the GEM of each scRNA-seq dataset and then transformed to the DM. Due to a large number of dropout events (i.e., zero values) in scRNA-seq data, we filtered out the genes expressed in less than ten cells and performed log_2_ transformation with a pseudo count of one on the raw matrix, i.e., raw matrix → GEM → SCNs → DM. Therefore, the DM has the same number of columns and rows as the GEM.

**Fig. 2:**
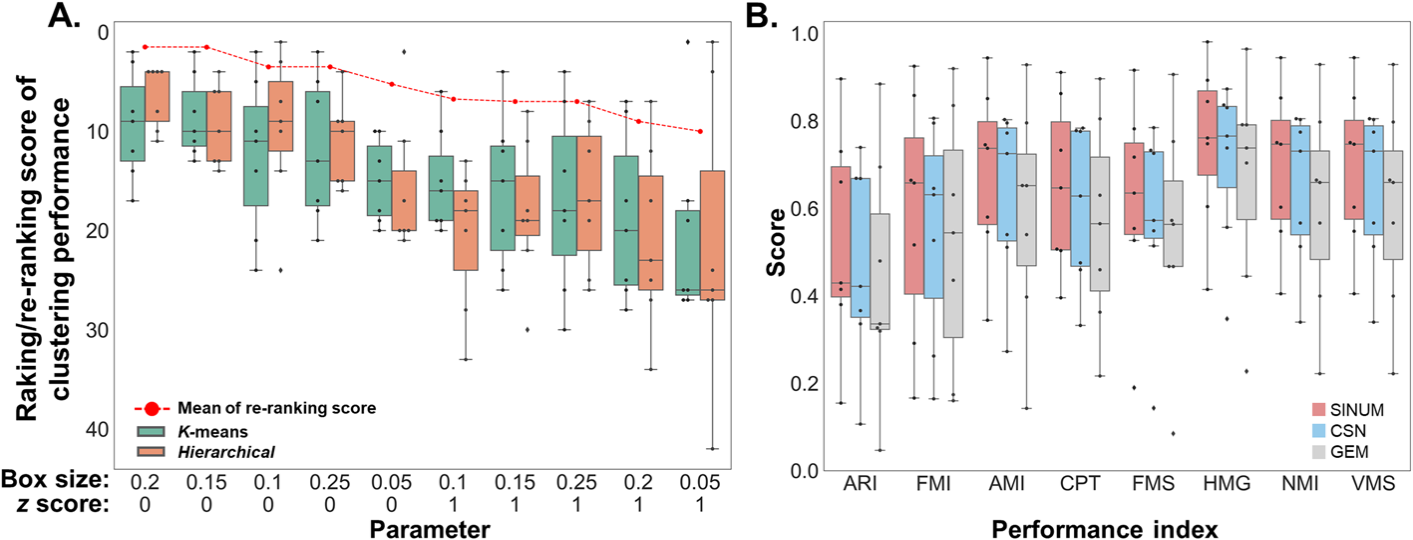
Clustering performances of DMs and GEMs on seven scRNA-seq datasets. **A** Distributions of ranking/re-ranking scores of clustering performances of SINUM DMs generated at different parameter combinations (on the *x*-axis, sorted by decreasing average re-ranking scores). In this analysis, we utilized the FEAST algorithm to select the top 1000 representative genes for each dataset and further performed the SINUM method to build SCNs and DMs. The *k*-means (green) and hierarchical (orange) clustering performances of these SINUM DMs on seven datasets (dots) were shown in the boxplot. The red dashed line represents the mean of the re-ranking scores evaluated by the ranking scores across seven datasets using two clustering methods. For each dataset, the ranking scores were measured by sorting the clustering performances at different parameter combinations between various box sizes and *z* score thresholds (for details, see Additional file 1: Tables S3 and S4). **B** Comparisons of *k*-means clustering performances of SINUM DMs (red), CSN DMs (blue), and GEMs (gray) on seven scRNA-seq datasets, evaluated by eight distinct performance indexes. Note that the SCNs were generated using whole GEMs. Each dot presents a dataset. ARI, adjusted rand index; FMI, F-measure index; AMI, adjusted mutual information; CPT, completeness scores; FMS, fowlkes-mallows scores; HMG, homogeneity scores; NMI, normalized mutual information; VMS, V-measure scores

The SINUM method possesses a certain degree of unavoidable time complexity because of computing $m\left( {m - 1} \right)/2$ pair of genes when given a total of *m* genes. Thus, we first applied the FEAture SelecTion (FEAST) tool [30] to select the top 1,000 significant features from the raw matrix as representative genes for determining the suggested setting of two adjustable parameters in the SINUM method (i.e., box size and z-score threshold). The FEAST tool, designed for feature selection of scRNA-seq, can find clusters with high confidence by consensus clustering method, retains cells with high correlation with clusters, and finally identifies the significant features through F-statistics and ranking. The suggested parameters were determined by the ranking and re-ranking scores of the clustering performances based on the selected 1,000 genes (Fig. 2A). Specifically, we measured the ranking score for each dataset by sorting their F-measure scores for SINUM DMs in descending order using 25 different parameter combinations, including five different box sizes (0.05, 0.1, 0.15, 0.2, 0.25) and five different z-score thresholds (− 2,− 1, 0, 1, 2). Next, the re-ranking score for each parameter combination using k-means (or hierarchical clustering) was evaluated based on the mean of ranking scores across seven datasets. Finally, the re-ranking scores from two clustering methods were averaged to determine the overall performance. Since the smaller mean of re-ranking scores represents better performance, we used the box size = 0.2 and z-score > 0 as default parameters to build SINUM SCNs in this study.

### ScRNA-seq datasets for validation

To validate and compare SINUM SCNs, CSN SCNs, and GEM, we collected and downloaded seven ScRNA-seq datasets from the Gene Expression Omnibus (GEO) database [31]. The annotations of corresponding cell types provided by original works were assembled for each dataset. The brief introductions and summaries of these seven datasets are described as follows and listed in Additional file 1: Table S2.

*Chu-type dataset* [32], including seven cell types and 1,018 cells, comprises the cells of human embryonic stem cell-derived lineage-specific progenitors. The cell types contain H1 embryonic stem cells, H9 embryonic stem cells, human foreskin fibroblasts, neuronal progenitor cells, definitive endoderm cells, endothelial cells, and trophoblast-like cells.

*Chu-time dataset* [32], including six cell types and 758 cells, is composed of cells from human embryonic stem cells to produce definitive endoderm cells at different time points. The cell types contain six different time points, including 0 h, 12 h, 24 h, 36 h, 72 h, and 96 h of differentiation.

*Haring dataset* [33], including 30 cell types and 1,545 cells, is composed of mouse brain dorsal horn cells. The cell types contain 15 inhibitory and 15 excitatory neuronal types, including the Gaba 1–15 and Glut 1–15, revealed by clustering cells with similar characteristics.

*Baron dataset* [34] has six sub-datasets, including four human and two mice samples, the human sample 1 was collected and used in this study. The human sample 1 sub-dataset, containing 14 cell types and 1,937 cells, comprises individual pancreatic cells from one of four human donors. The cell types are composed of alpha, beta, delta, gamma, epsilon, acinar, ductal, quiescent stellate, activated stellate, endothelial, macrophage, mast, cytotoxic T, and Schwann cells.

*Romanov dataset* [35], including seven cell types and 2,881 cells, is composed of the mouse neuron cells of the central column of the medial-ventral diencephalon. The cell types contain oligodendrocytes, astrocytes, ependymal cells, microglial cells, endothelial cells, vascular and smooth muscle lineage cells, and neurons.

*Yan dataset* [4, 36], including five cell types and 124 cells, comprises the cells from human pre-implantation embryos and human embryonic stem cells. The cell types contain human embryonic stem cells and different stages of human preimplantation blastomere, containing oocyte, zygote, 2-cell, late developmental cells.

*Darmanis dataset* [37], including nine cell types and 466 cells, comprises the cortex cells from adult and fetal human brain samples. The cell types contain astrocytes, neurons, oligodendrocytes, endothelial cells, oligodendrocyte precursor cells (OPCs), replicating neuronal progenitors (fetal- replicating), quiescent newly born neurons (fetal-quiescent).

## Results

### Single-cell network using mutual information (*SINUM*)

Figure 1 illustrates the process of implementing the SINUM method to infer the SCNs with the MI score for each edge by quantifying the mutual dependence between the two genes. Given gene expression data of a population of cells, the aim of the SINUM method is to model SCNs for better estimating network-level similarity and diversity at a single-cell resolution level. To optimally model the SCNs, the SINUM method comprises two adjustable parameters, the box size to define the neighborhood of the expression distribution between any two genes across cells and the *z* score threshold for determining a significant level of mutual dependence for each edge. In other words, the box size is used to define the upper and lower boundaries of the target cell in the scatter diagram for every two genes, which represents the neighborhoods of the target cell for the calculation of entropy (*H*) and MI score. To cover the same number of cells, the neighborhood will be changed to smaller and larger when the target cell was located in the dense and sparse areas of the scatter diagram, respectively. If the *z* score is larger than the threshold, there is a correlation between genes *X* and *Y* for the target cell *c*, represented by an edge; otherwise, there is no edge. Finally, the DM was constructed by counting the number of edges connected to every gene in each SCN.

To determine the suggested parameter combination for our SINUM, we first collected seven scRNA-seq datasets given with the cell type labels from the GEO database. According to *k*-means and hierarchical clustering analyses of the DMs for the top 1,000 representative genes at different parameter combinations (a total of 25), we found that the SINUM DMs achieved the highest mean F-measure scores (0.595) on these seven datasets when the box size and *z* score threshold were set to 0.2 and 0, respectively (Additional file 1: Fig. S1 and Tables S3 and S4). Moreover, the mean F-measure scores for SINUM DMs using this parameter combination were also higher than those for CSN DMs (< 0.561) and GEM (< 0.565), irrespective of matrix size (i.e., 1000 or all genes). Additionally, to avoid clustering performances being dominant by certain datasets, we further measured the ranking and re-ranking scores for determining the suggested parameters (see the details in Materials and methods). Similar to the above results, the result showed that the SINUM DM generated by using box size = 0.2 and *z* score > 0 has a smaller mean of re-ranking scores between two clustering methods than other parameter combinations (Fig. 2A). Note that the smaller the mean of re-ranking scores, the better the recommendation. Thus, the box size = 0.2 and *z* score > 0 as default parameters were used to build SINUM SCNs.

### Comparison of clustering performances for SINUM DMs, CSN DMs, and GEMs using the whole gene expression profiles of seven datasets

**Fig. 3:**
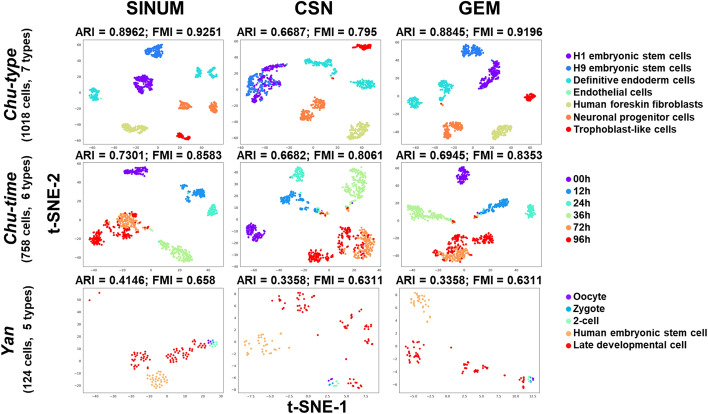
t-SNE plots for visualizing the *k*-means clustering performances of SINUM DMs, CSN DMs, and GEMs on *Chu-type*, *Chu-time*, and *Yan* datasets. For visualization, we applied t-distributed stochastic neighbor embedding (t-SNE) to reduce the dimensions to two after reducing by principal component analysis (PCA) to 20 dimensions. The *x*-axis and *y*-axis in each plot represent t-SNE-1 and t-SNE-2, respectively. For each dataset, distinct cell types are marked by different colors. Adjusted rand index (ARI) and F-measure index (FMI) were employed in comparison of *k*-means clustering performances since the cell type labels of each dataset had been known

It has been known that identifying cell types by inputting global transcriptome profiles into unsupervised clustering is one of the main purposes of scRNA-seq [9, 38]. Thus, we first constructed the SINUM DMs, CSN DMs, and GEMs based on whole gene expression profiles of seven datasets. The eight performance indexes (e.g., ARI and FMI) were then used for performance evaluation, according to the known cell type labels of each dataset. The results indicated that the clustering performances for SINUM DMs were superior to CSN DMs and GEM irrespective of performance index or clustering method, except for the *Darmanis* dataset (Fig. 2B and Additional file 1: Table S5). Based on the t-SNE plots, we observed that different cell types could be distinguished more clearly by SINUM DMs than by CSN DMs and GEM (Fig. 3 and Additional file 1: Figs. S2 and S3); for example, H1 and H9 embryonic cells on the *Chu*-type dataset and cells captured at 0, 12, 24, and 36 h of differentiation on *Chu*-time dataset. These results validate the effectiveness of the SINUM method in presenting a view of biological network systems to discriminate the cell types.

### Characteristics of SCNs constructed by different methods

**Fig. 4:**
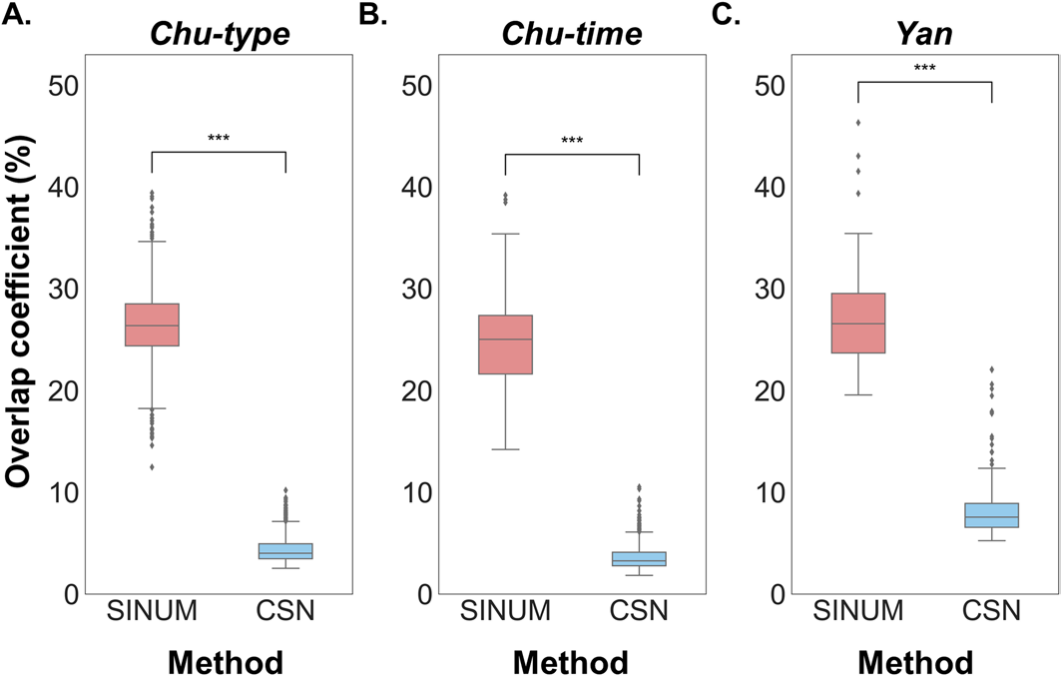
Distribution of overlap coefficient between the edges of STRING human PPI network and the SINUM (red) or CSN (blue) SCNs on (**A**) *Chu-type*, (**B**) *Chu-time*, and (**C**) *Yan* datasets. The overlap coefficient is defined as the size of the intersection divided by the smaller of the size of the two edge sets between the STRING human PPI network and each SCN. Data between the different methods were statistically analyzed using Wilcoxon signed-rank test. A triple asterisk indicates the *p* value < 0.005

The interactions between genes/proteins are considered the backbone of the cellular networks to regulate most biological processes [39]. To validate the identified edges of SCNs generated by SINUM and CSN methods, we first assembled two human PPI networks from the STRING database [25], containing 12,151 proteins (nodes) and 318,125 PPIs (edges), and from 21 public databases curated by Gysi et al*.* [26], including 18,505 proteins and 327,924 PPIs. Next, we constructed the SINUM and CSN SCNs based on the default parameters using seven scRNA-seq datasets and calculated the overlap coefficient between the STRING (or Gysi) PPI network and each of either SINUM or CSN SCNs (Fig. 4 and Additional file 1: Table S1). The results showed that the overlap coefficients of SINUM SCNs are significantly higher than CSN ones (*p* < 0.005, Wilcoxon signed-rank test), suggesting that SINUM has an advantage over CSN in inferring high-confidence edges of networks at a single cell level.

**Fig. 5:**
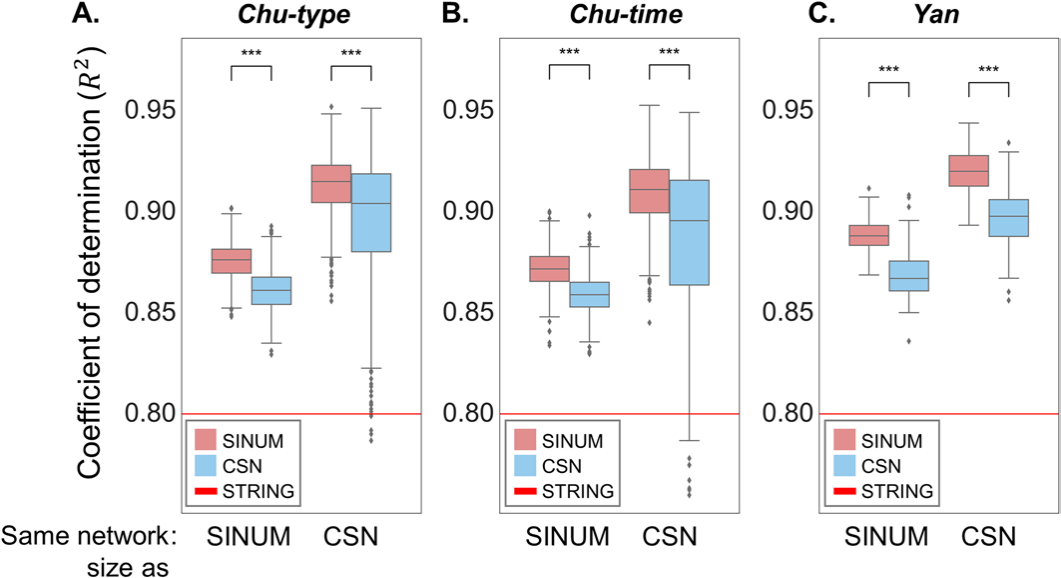
Distribution of coefficient of determination ($R^{2}$) values for the SINUM (red) and CSN (blue) SCNs constructed using (**A**) *Chu-type*, (**B**) *Chu-time*, and (**C**) *Yan* datasets. To avoid network size bias, each SCN was generated by SINUM (or CSN) method via selecting the edge sorted by respective confidence scores and had the same edge number (i.e., same network size) as the original CSN (or SINUM) SCN. Next, these SCNs were intersected with the STRING human PPI network to evaluate the scale-free topology fit index (i.e., $R^{2}$ value). The red line represents the $R^{2}$ value for the STRING human PPI network

Recently, it has been an important finding in studying cellular architecture that most biological networks exhibit a scale-free characteristic, $P\left( k \right)\sim k^{ - \gamma }$ [40, 41], where $\gamma$ is the degree exponent; the probability of a node with *k* links decreases as the node degree increases on a log–log plot (i.e., a power-law distribution). The coefficient of determination (*R*^*2*^) was commonly used to assess the ability of the fitted line to accurately describe degree distributions with scale-free network properties [i.e., the linearity between log(*P*(*k*)) and log(*k*)] [16, 42]. To further assess whether SCNs are likely to exhibit a scale-free topology, we adjusted the network scales (i.e., same edge number) by the SINUM and CSN SCN sizes separately to avoid a bias caused by network sizes (Additional file 1: Figure S4). First, for adjusting the network scale by each SINUM SCN size, we extracted the overlapping subnetwork (i.e., intersection) between each SINUM SCN and the STRING human PPI network. Next, the edge *z* scores of CSN were assigned to all the edges of the STRING human PPI network. Finally, according to the number of edges in each overlapping subnetwork of SINUM, we further selected the same edge number from the STRING human PPI network in descending order of edge *z* scores of CSN to build the corresponding subnetwork of CSN. In other words, SINUM and CSN subnetworks have the same edge number but may have different network topologies. The same procedure was also used to adjust the SINUM network scale by each CSN SCN size. According to the results, SINUM SCNs showed higher *R*^*2*^ values than CSN ones on several datasets regardless of network scale (*p* < 0.005, Wilcoxon signed-rank test; Fig. 5). Additionally, the higher $\gamma$ values come from SINUM SCNs depending on SINUM’s network scales (*p* < 0.005, Wilcoxon signed-rank test; Additional file 1: Fig. S5). With CSN’s network scales, despite higher γ values for CSN SCNs, the variations of γ values among CSN SCNs on different datasets are apparently higher than those among SINUM SCNs. Moreover, compared to the original STRING PPI network, we also observed that the overlapping networks between the STRING PPI network and SINUM SCNs are more likely to fit scale-free characteristics (i.e., higher *R*^*2*^ and $\gamma$ values), especially in *R*^*2*^ values (Fig. 5 and Additional file 1: Fig. S5). Interestingly, we found that the $\gamma$ values for most SINUM and CSN SCNs are between 1 and 2; it is reminiscent of the concordance for many biological networks reported by the previous studies [13, 25, 26, 43]. Taken together, these results implied that our SINUM method could detect high-confidence edges and build the SCNs with the characteristic of scale-free networks.

### Detection of the cell-type marker genes and gene pairs

**Fig. 6:**
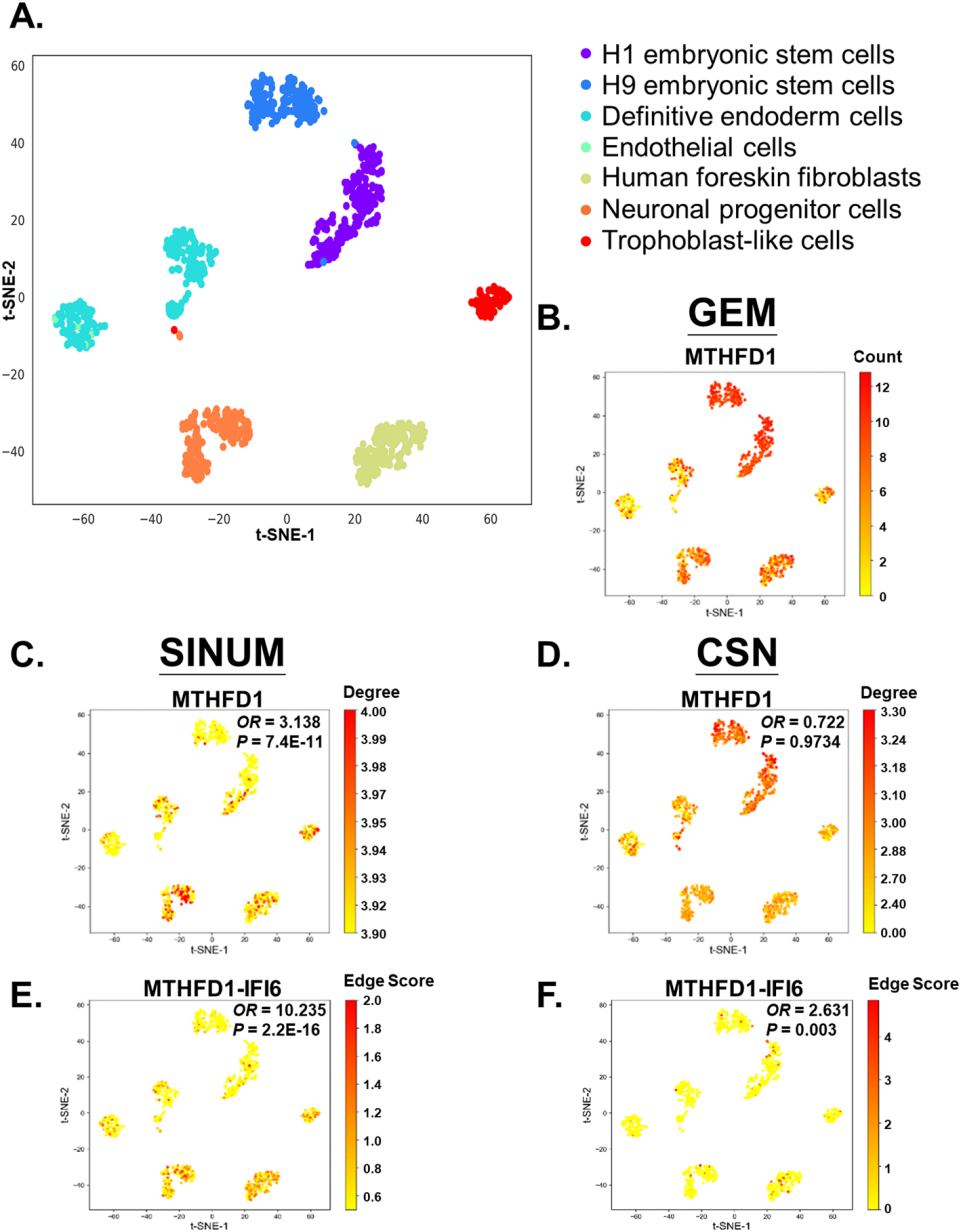
Detection comparison of cell-type markers in human foreskin fibroblast (HFF) using the SINUM DMs, CSN DMs, and GEMs based on *Chu-type* dataset. (**A**) t-SNE plot of GEM for *Chu-type* dataset, colored by cell types (ARI = 0.89 and FMI = 0.92). The cell type of HFF (green) was chosen to detect the potential markers using the SINUM DMs, CSN DMs, and GEMs, such as gene *MTHFD1* (methylenetetrahydrofolate dehydrogenase 1) and gene pair *MTHFD1*-*IFI6* (interferon alpha inducible protein 6). Gene *MTHFD1* in the t-SNE plots, colored by (**B**) the gene expression level and the network degree level in the (**C**) SINUM SCNs and (**D**) in the SINUM SCNs. The degree *d* for networks and raw count *r* for gene expression were transformed by log_10_(*d* + *1*) and log_2_(*r* + *1*), respectively. Fisher's exact test was performed to statistically test whether the proportion of gene *MTHFD1* as a hub in SCNs is higher among the HFF cells than among the other cells. The nodes with degrees within the top 25% of all nodes were defined as the hubs (i.e., hub genes) of each SCN. The odds ratio (*OR*) and *p* value (*P*) of statistical analysis are shown. Edge *MTHFD1-IFI6* in the t-SNE plots, colored by the (E) SINUM edge scores (i.e., *z* scores) and (F) CSN edge scores (i.e., *z* scores). Fisher's exact test was performed to statistically test whether the proportion of edges in SCNs is higher among the HFF cells than among the other cells

The previous work has indicated that some genes (also called ‘dark’ genes) have no significant difference at a gene expression level but at a network degree level between a specific cell type and the other cell types [19]. These genes have been suggested as the signatures in distinguishing a specific cell type. Thus, we further examined whether the network-based methods (i.e., SINUM and CSN) could identify cell-type marker genes and gene pairs on the *Chu*-type dataset, including H1 embryonic stem cells, H9 embryonic stem cells, definitive endoderm cells, endothelial cells, human foreskin fibroblasts (HFF), neuronal progenitor cells (NPC), and trophoblast-like cells (Fig. 6A and Additional file 1: Fig. S6A). Based on the comparison between each cell type and the others, we then determined the cell-type marker genes and gene pairs (Additional file 1: Note S2). Obviously, the genes *MTHFD1* (encoding methylenetetrahydrofolate dehydrogenase 1; Fig. 6C) and *RIN2* (encoding Ras and Rab interactor 2; Additional file 1: Fig. S6C) exhibit higher degrees in SINUM SCNs of HFF and NPC types than those in the other cell types, respectively. However, between the corresponding cell types and the others, these two genes have relatively small differences in network degrees of CSN SCNs and in gene expression (Fig. 6B, D and Additional file 1: Fig. S6B, D).

We next statistically tested whether the proportion of gene *MTHFD1* (or *RIN2*) as a hub in SCNs is higher among the HFF (or NPC) cells than among the other cells, where the nodes with degrees within the top 25% of all nodes were defined as the hubs (i.e., hub genes) of each SCN [44]. The results showed that gene *MTHFD1* is more likely to be the hubs of SINUM SCNs in HFF cells [*p* value = 7.4e−11 and odds ratio (*OR*) = 3.14; Fisher's exact test] than those of CSN SCNs (*p* value = 0.97 and *OR* = 0.72; Fig. 6C, D). Additionally, we also found that the proportion of edge *MTHFD1-IFI6* (interferon alpha inducible protein 6) in SINUM SCNs is significantly higher among the HFF cells than among the other cells (Fig. 6E, F). In HFF cells, the gene *MTHFD1* is mainly expressed in the G1/S and G2 phases of the cell cycle in HFF cells and plays a regulatory role in the early stage of cell growth [45, 46]. Similar results were observed for gene *RIN2* and gene pair *RIN2-WIPF3* (WAS/WASL-interacting protein family member 3) in NPC cells (Additional file 1: Fig. S6C-F). These observations are reminiscent of the findings of the dark genes between normal and case samples [47] and the non-coding RNAs as dark matter in sequence [48]. In short, our SINUM has the ability to characterize cell-type marker genes and gene pairs, which may play important roles in specific cell types but are generally neglected by traditional differential gene expression analysis.

### Network rewiring during embryo development at a single-cell level

**Fig. 7:**
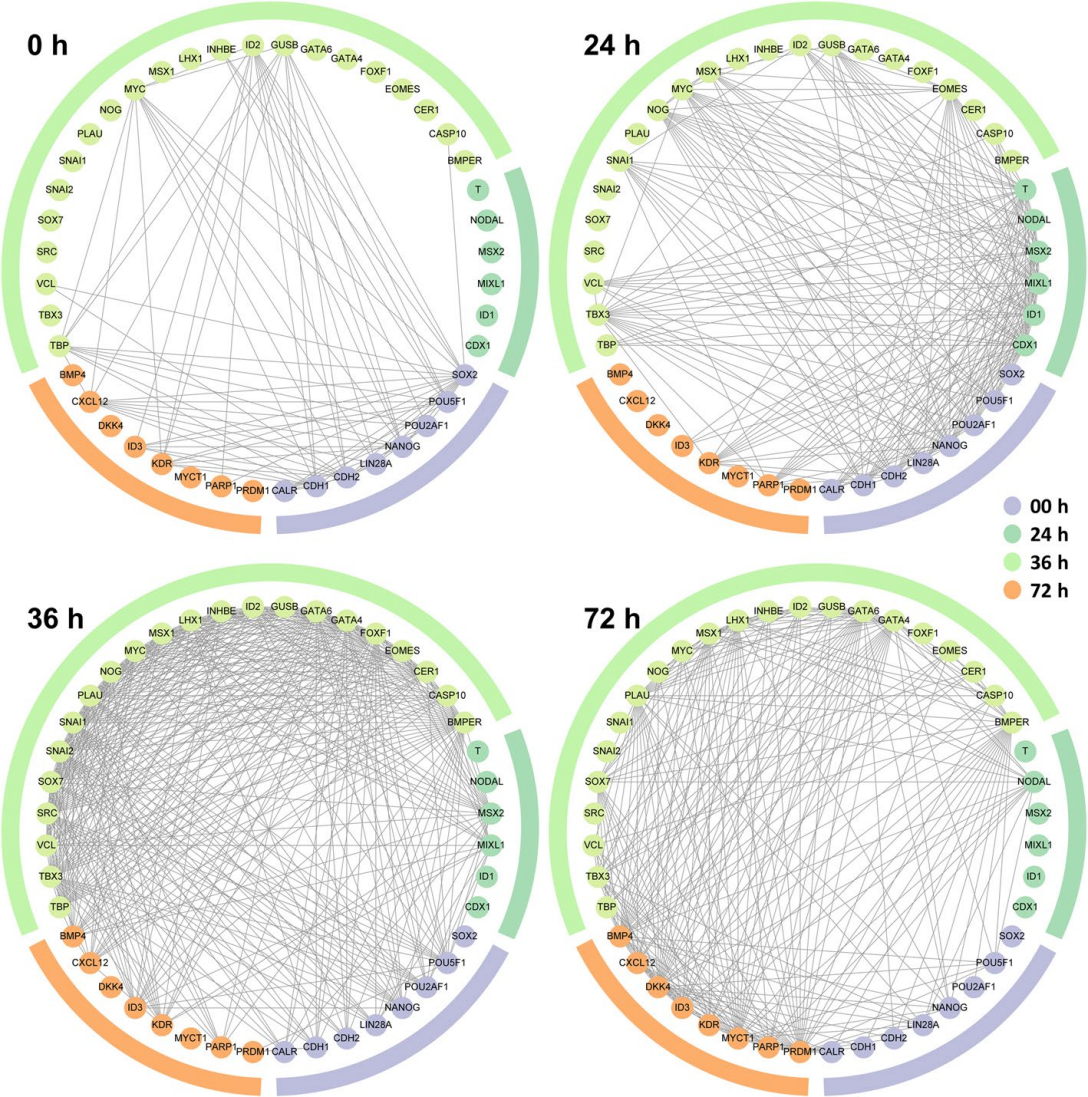
Network rewiring diagrams among 44 embryo development-related genes at four different time points based on the *Chu-time* dataset. The SCNs were constructed by our SINUM method using the *Chu-time* dataset. Each edge in the diagram indicates that two genes are associated in more than 70% of cells at a particular time point. The node color (or colored outline) indicates that the gene has the highest mean degree among all SCNs (i.e., all cells) at a specific time point than at the other time points

The indispensable vital organs in adults, such as the trachea, lungs, liver, pancreas, and thyroid, are all derived from the definitive endoderm [32, 49, 50]. Here, we applied SINUM to reconstruct time-dependent networks on the *Chu-time* dataset for exploring embryo development at a single-cell resolution. The dataset comprises 758 cells derived from human embryonic stem cells that give rise to definitive endoderm cells at six time points (0, 12, 24, 36, 72, 96 h). We then collected 58 embryo development-related genes (Additional file 1: Table S6) from several studies [19, 32, 51, 52] and performed an analysis of time-dependent network rewiring on the basis of these genes. Note that 4 out of 58 genes are not expressed or expressed in a small number of cells (< 10 cells) on the *Chu-time* dataset and would be discarded in this analysis. Each edge in a time-dependent network represents that the two genes were determined as associated by our SINUM method in more than 70% of cells at a particular time point. We found that 81.5% of genes (44/54) have at least an association with each other at one of these time points (Fig. 7). The results illustrated that the edge density of the network gradually increased until the 36th hour and then decreased, implying that the associations among these 44 genes were strongest at the 36th hour (Fig. 7). Consistent with our result, previous research demonstrated that the markers of mesodermal and endodermal lineage were significantly up-regulated within 36 h after differential induction of human embryonic stem cells (hESC) [53], implicating the 36th hour is a critical stage of embryo differentiation. Moreover, the 36th hour is a key time point during the progression to the four-cell stage in the human embryo development, as well as the activation point of ESSP2, which refers to a set of embryonic activated genes [54]. Collectively, all evidence indicates that the SINUM method not only successfully identifies the important time points of hESC differentiation process but also provides potential associations among differentiation-related genes.

**Fig. 8:**
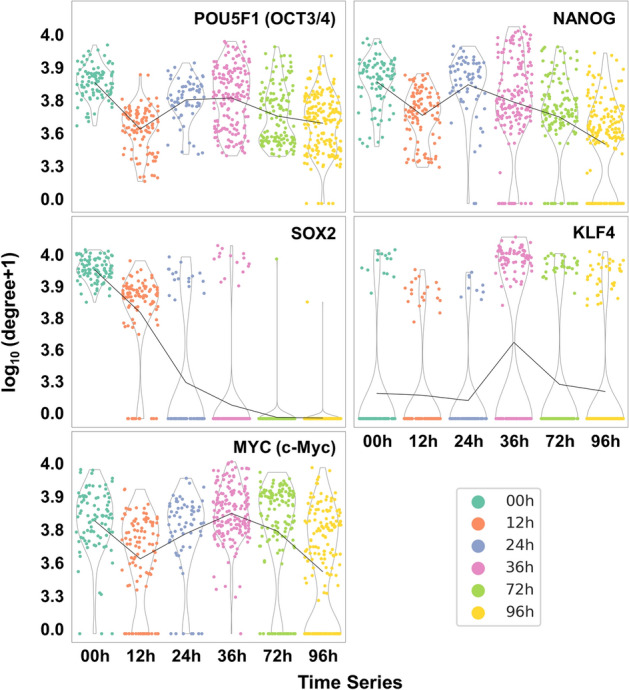
Violin plots of network degrees for five key transcription factors involved in the induction and maintenance of pluripotency in embryonic stem cells. The network degrees of the five key transcription factor genes, including *POU5F1* (*OCT3*/*4*), *NANOG*, *SOX2*, *KLF4*, and *MYC* (*c-Myc*), in the SINUM SCNs generated using the *Chu-time* dataset are shown at six time points during embryo development. The dots with the same color represent the network degrees in different SINUM SCNs inferred at each time point. The degree (*d*) was transformed by log_10_(*d* + *1*). The black line indicates the mean degree of the genes among all SCNs at that time point

POU5F1 (OCT3/4), MYC (c-Myc), SOX2, and KLF4 have been recognized as key transcription factors during pluripotent stem cell induction [55]. Also, recent works indicated that NANOG as a transcription factor plays a crucial role in self-renewal and maintenance of pluripotency in hESC [56]. On the basis of these five transcription factor genes, we executed the network degree analysis of SINUM SCNs built using the *Chu-time* dataset. The results showed that genes *POU5F1*, *NANOG*, and *SOX2* exhibited the highest network degree at 0 h, suggesting these genes play a critical regulatory role in the early stage of embryo development (Fig. 8). We further found that the highest degree of genes *MYC* and *KLF4* occur at the 36th hour. Previous studies have observed that the gene *KLF4* is highly expressed from mouse four-cell to morula stage embryos and in human morula stage embryos [57]. Moreover, gene *MYC* has been indicated to be highly expressed from the eight-cell to blastocyst stage of embryo development in both mice and humans [55, 58]. These results suggest that *MYC* and *KLF4* may regulate and induce the embryos into the next developmental stage after activation of *POU5F1*, *NANOG*, and *SOX2*. Taken together, the network degree analysis using our SINUM enables the characterizations of time-dependent network rewiring corresponding to embryo developmental stages at a single-cell resolution.

## Discussion

The scRNA-seq technology provides the gene expression data at a single-cell resolution, but extracting inherent biological system information (e.g., cellular networks) to get insight into the detail remains a major challenge. In this study, we provide SINUM as a new strategy for single-cell data analysis, which introduces mutual information to reconstruct SCNs. SINUM has several unique advantages. First, without prior knowledge of clusters or cell types, applying the dimension-reduction and clustering methods in the network degree matrix of SINUM SCNs is able to identify cell types on various scRNA-seq data and is superior to those of the other methods. Previous work has also mentioned that MI is relatively robust in terms of distinguishing various clustering solutions [59]. Second, capturing the intrinsic network architecture of each cell from scRNA-seq data can be considered a reverse engineering task. Compared to traditional statistical measures, the reverse engineering task will benefit from the flexibility of information theoretical measures [60]. The accurate estimate of probability distributions for calculating MI generally depends on the sample sizes [23, 24]. Therefore, our theoretical model using density estimation and data discretization to conduct grid-based MI measures could be expected to be sensitive and appropriate for predicting gene–gene associations from scRNA-seq data, which are generally sufficiently large. Additionally, the SINUM method is designed to construct a close-to-real network (i.e., a network with scale-free topology) on a single-cell basis from RNA-seq data; in other words, reconstructing the cellular network for each cell, including both cell-specific and common edges. These may explain why SINUM SCNs overlap with the two human PPI networks more than CSN SCNs since these PPI networks themselves also displays a scale-free feature (*R*^*2*^ value of 0.81 and γ value of 1.4 for STRING and *R*^*2*^ value of 0.88 and γ value of 1.6 for Gysi et al*.*). Third, our SINUM could identify cell-type marker genes and gene pairs with a differential network degree between a certain cell type and the others but no differential gene expression, such as *MTHFD1* and *MTHFD1-IFI6* in HFF cells. Finally, the SINUM method has a utility in investigating time-dependent changes in associations among functional genes and further compensates for the traditional differential expression analysis on scRNA-seq data.

SINUM using mutual information has several limitations and challenges relative to dynamical system models [61, 62]. First, our SINUM method may overestimate some indirect gene–gene associations if these two genes are directly associated with one or several common genes (i.e., intermediary genes). Therefore, one of our future topics is to reduce the false positive rate since the current SINUM SCNs may have higher density than the real molecular network (e.g., gene regulatory network or PPI network) in the target cell. Second, similar to the CSN method, our SINUM method was designed to detect gene–gene associations (undirected edges) for building single-cell networks. In biological networks, the directions of the edges in many pathways, such as signaling transduction, metabolic reaction, and transcriptional/translational regulation, could be regarded as the causal relationships between two molecules and are important to understand cellular functions and processes as well as disease mechanisms. Hence, in the future, we aim to determine the directions between two genes in SCNs. For example, partial mutual information may be a useful means for detecting candidate causal and direct interactions [60]. Finally, the computational time and memory usage of our SINUM method grow exponentially with the numbers of genes and gene pairs (Additional file 1: Fig. S7). It is reminiscent of the discussion of scalability issues regarding the feature dimension and the sample size for single-cell data analysis in previous work [63]. Indeed, the SINUM method could be classified as a multivariate gene/biomarker filtering method for scRNA-seq data analysis due to transforming the gene expression matrix into a network degree matrix for feature transformation and selection. Thus, SINUM suffers from the same problem of the scalability of the feature selection, including the feature dimensionality (i.e., gene/gene pair number) and the sample size (i.e., cell number). There are two possible solutions: (1) parallel computing to reduce the computational time; and (2) only estimating the gene pairs which have the corresponding edges in the reference network (e.g., the STRING or literature-curated PPI network). For example, the computational time and the memory usage of the SINUM method in detecting the same edge number as the STRING human PPI network are around 0.4 min and 35 MB per cell, respectively. Therefore, we collected a large scRNA-seq dataset, *10* × *PBMC*, with more than 10 k cells and used solution (2) to ease the computational burden for examining whether SINUM still outperforms the CSN method in clustering performances. Based on the reference network (here is the STRING human PPI network), we constructed SINUM and CSN DMs using *10* × *PBMC* and the other seven datasets and then conducted the dimensionality-reduction and clustering analysis. Similar to the above results using the whole gene expression profiles, we found that SINUM DMs achieved better performances than CSN DMs on most of the datasets regardless of sample size (i.e., cell number) or evaluation indexes (Additional file 1: Table S7). This analysis suggests the feasibility of the SINUM method using a reference network to reduce computational time while preserving the expected performance.

## Conclusion

Our results shed light on the availability of MI measures in constructing SCNs and validated the accuracy (e.g., adjusted rand index and F-measure index) and robustness (e.g., datasets with different cell numbers) of SINUM in cell type identification. To our knowledge, SINUM provides a new framework to facilitate the discovery of network architecture at a single-cell level, heterogeneity among/within cell populations, as well as cell type-specific markers.

## Supplementary Information

Additional file 1

## Data Availability

Codes for SINUM are freely available at https://github.com/SysMednet/SINUM. This research used the publicly available datasets published by other researchers as cited in the manuscript.

## Abbreviations

AMI: Adjusted mutual information
ARI: Adjusted rand index
CPT: Completeness scores
CSN: Cell-specific network
DM: Degree matrix
FEAST: Feature selection
FMI: F-Measure index
FMS: Fowlkes–Mallows scores
GEM: Gene expression matrix
GEO: Gene expression omnibus
hESC: Human embryonic stem cells
HFF: Human foreskin fibroblasts
HMG: Homogeneity scores
*IFI6*: Interferon alpha inducible protein 6
MI: Mutual information
*MTHFD1*: Methylenetetrahydrofolate dehydrogenase 1
NMI: Normalized mutual information
NPC: Neuronal progenitor cells
PCA: Principal component analysis
PIDC: Partial information decomposition and context
PPI: Protein‒protein interaction
*RIN2*: Ras and Rab interactor 2
SCN: Single-cell network
SINUM: Single-cell network using mutual information
t-SNE: t-Distributed stochastic neighbor embedding
*WIPF3*: WAS/WASL-interacting protein family member 3
