# Supplementary material for: Inference of single-cell network using mutual information for scRNA-seq data analysis
Source: BMC Bioinformatics. 2024 Sep 5;25(Suppl 2):292. doi: 10.1186/s12859-024-05895-3 (PMC11378379; doi:10.1186/s12859-024-05895-3)
Supplement: Supplementary file 1 — Additional file 1 [file 12859_2024_5895_MOESM1_ESM.docx]

**Supplementary Information**

**Inference of single-cell network using mutual information for** **scRNA-seq data analysis**

Lan-Yun Chang^1,†^, Ting-Yi Hao^1,†^, Wei-Jie Wang^1,†^, and Chun-Yu Lin^1,2,3,4,5,6,*^

^1^Institute of Bioinformatics and Systems Biology, National Yang Ming Chiao Tung University, Hsinchu 300, Taiwan, ^2^Department of Biological Science and Technology, National Yang Ming Chiao Tung University, Hsinchu 300, Taiwan, ^3^Institute of Data Science and Engineering, National Yang Ming Chiao Tung University, Hsinchu 300, Taiwan, ^4^Center for Intelligent Drug Systems and Smart Bio-devices, National Yang Ming Chiao Tung University, Hsinchu 300, Taiwan, ^5^Cancer and Immunology Research Center, National Yang Ming Chiao Tung University, Taipei, 112 Taiwan and ^6^School of Dentistry, Kaohsiung Medical University, Kaohsiung 807, Taiwan

*To whom correspondence should be addressed.

^†^Lan-Yun Chang, Ting-Yi Hao1, and Wei-Jie Wang have contributed equally to this work

**Supplementary Notes**

**Note S1:** **Network degree matrix transformed from SCNs**

SINUM SCNs can be directly applied for different biological studies at a network level; nevertheless, the storage and memory sizes place an undeniable constraint when it comes to computational complexity. In addition, SCNs with different dimensionality other than the original GEM would lose plenty of opportunities for performance comparison in most of the subsequent scRNA-seq analyses. Therefore, we followed the previous studies [1, 2] to transform all SINUM SCNs to a DM, reflecting the network features, reducing the dimensionality, and retaining the same form as GEM simultaneously. Specifically, DM has the same dimension as the original GEM (i.e., *m* genes $\times$ *n* cells); each value in the DM represents the number of associations (edges) to other genes (nodes) for any given gene within each cell. In other words, for gene *X* in the network of cell *c* (**Fig. 1C**):

| ${DM}_{Xc}=\sum_{Y=1, Y\neq X}^{m} {edge}_{XY}^{(c)}$ | (S1) |
| --- | --- |

Moreover, we normalize the DM as suggested by Dai *et al.* [3] by

| $\hat{DM}_{Xc}=\frac{{DM}_{Xc}}{\sum_{i=1}^{m} {DM}_{ic}}\times\frac{a^{2}}{w}$ | (S2) |
| --- | --- |

where *w* is a constant and was suggested as 2,000 by Dai *et al*.; *a* is the average number of expressed genes per cell in the scRNA-seq data and is defined as

| $a=\frac{1}{n}\sum_{j=1}^{n} \sum_{i=1}^{m} sgn\left( {GEM}_{ij} \right)$ | (S3) |
| --- | --- |

Thus, each cell has the same sum of network degrees, which is decided by the mean number of genes with a non-zero value among *n* cells in each scRNA-seq dataset. The normalization is helpful in the comparison of the cells from different cell populations.

**Note S2: Identification of the cell-type marker genes and gene pairs**

Based on the comparison between each cell type and the others, we defined a cell-type marker gene that satisfies the following criteria: 1) log_10_(fold change) > 0.3 and *p* value of Mann–Whitney *U* test ≤ 0.01 at a network degree level and 2) |log_2_ (fold change)| < 1 and *p* value of Mann–Whitney *U* test > 0.1 at a gene expression level. Due to a large number of false zeros (i.e., dropout phenomenon) in scRNA-seq data, the non-zero ratio for each marker gene candidate in the specific cell type must be greater than 0.75. Moreover, a cell-type marker gene pair was determined based on the following criteria: 1) two genes were determined as an association by our SINUM in more than 50% of cells in the specific cell type, 2) fold change of edge scores is larger than 1.5, and 3) the *p* value of Mann–Whitney *U* test is less than 0.01.

**Supplementary Figures**


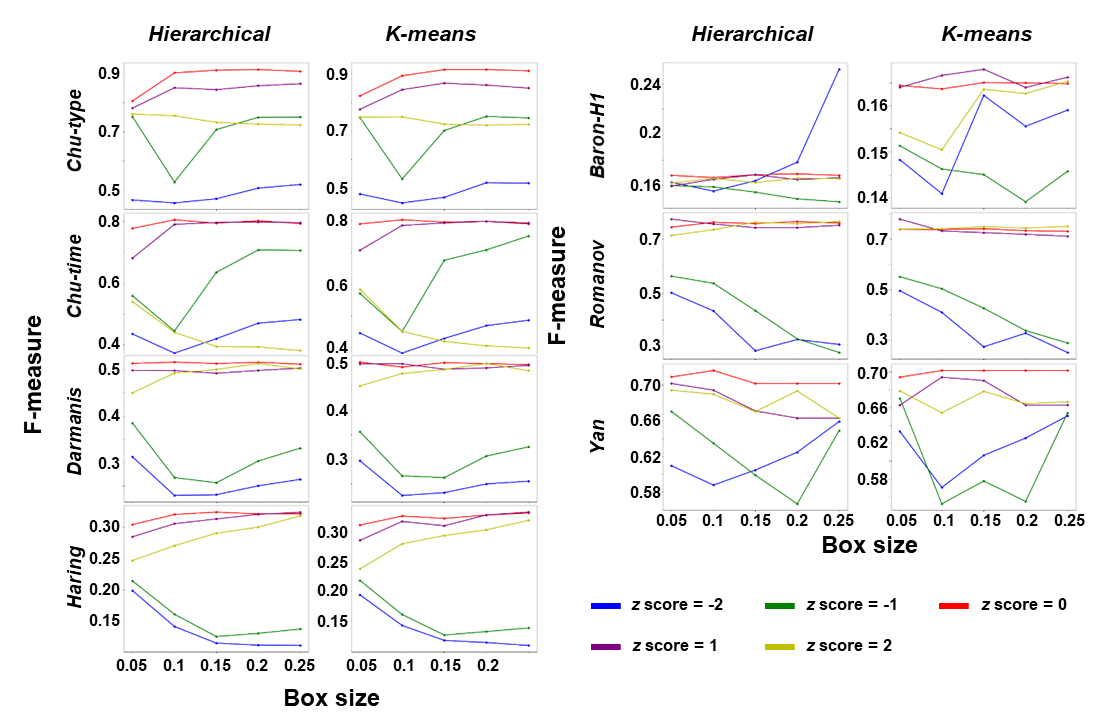


**Figure S1. Comparison of clustering performances using SINUM DMs generated at different parameter combinations on seven scRNA-seq datasets.**

In this analysis, we utilized the FEAST algorithm to select the top 1,000 representative genes (i.e., features) for each dataset and further performed the SINUM method to build SCNs and DMs. For these SINUM DMs, t-SNE was implemented to reduce the dimensions to two after reducing by PCA to 20 dimensions. The *k*-means and hierarchical clustering algorithms were performed in these SINUM DMs of seven datasets. The *x*- and *y*-axes represent box size and f-measure score, respectively. Different colored lines indicate different *z* score thresholds. Each value represents the median of clustering performances (repeated 30 times). For the SINUM method, systematic parameter variation provided evidence that the f-measure score more frequently achieved the highest median values across 25 parameter combinations when the box size and *z* score cutoff were set to 0.2 and 0, respectively.


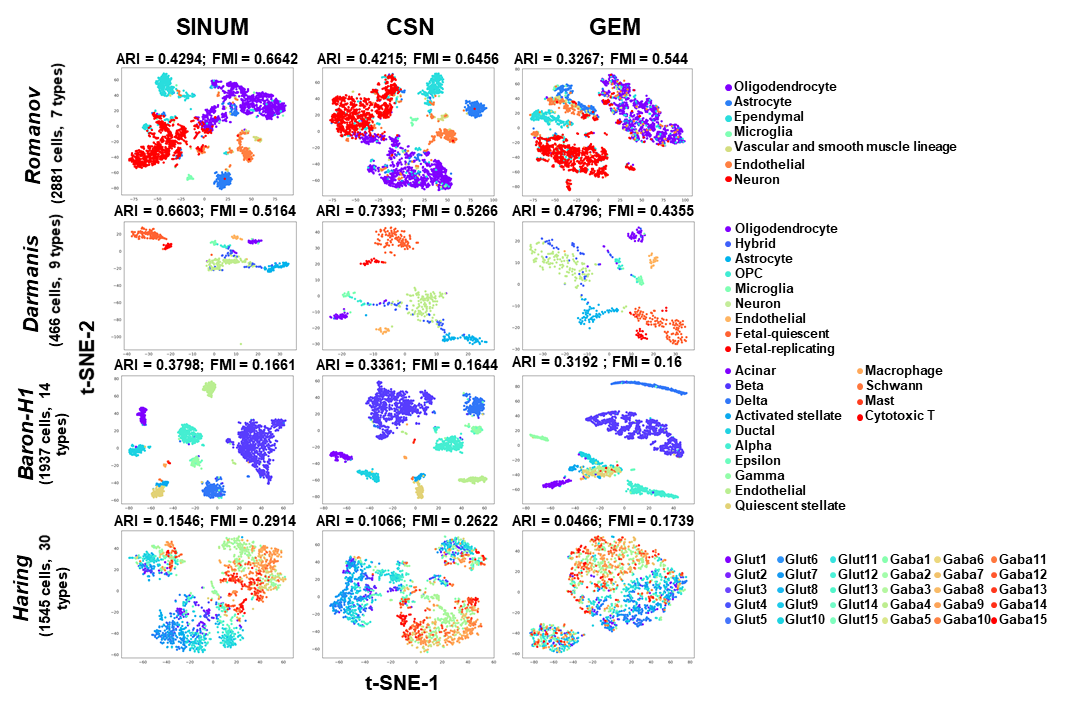


**Figure S2. t-SNE plots for visualizing the *k*-means clustering performances of SINUM DMs, CSN DMs, and GEMs on scRNA-seq datasets of *Romanov*, *Darmanis*, *Baron-H1*, and *Haring****.*

For visualization, we applied t-distributed stochastic neighbor embedding (t-SNE) to reduce the dimensions to two after reducing by principal component analysis (PCA) to 20 dimensions. The *x*-axis and *y*-axis in each plot represent t-SNE-1 and t-SNE-2, respectively. For each dataset, distinct cell types are marked by different colors. Adjusted rand index (ARI) and F-measure index (FMI) were employed in comparison of clustering performances since the cell type labels of each dataset had been known.


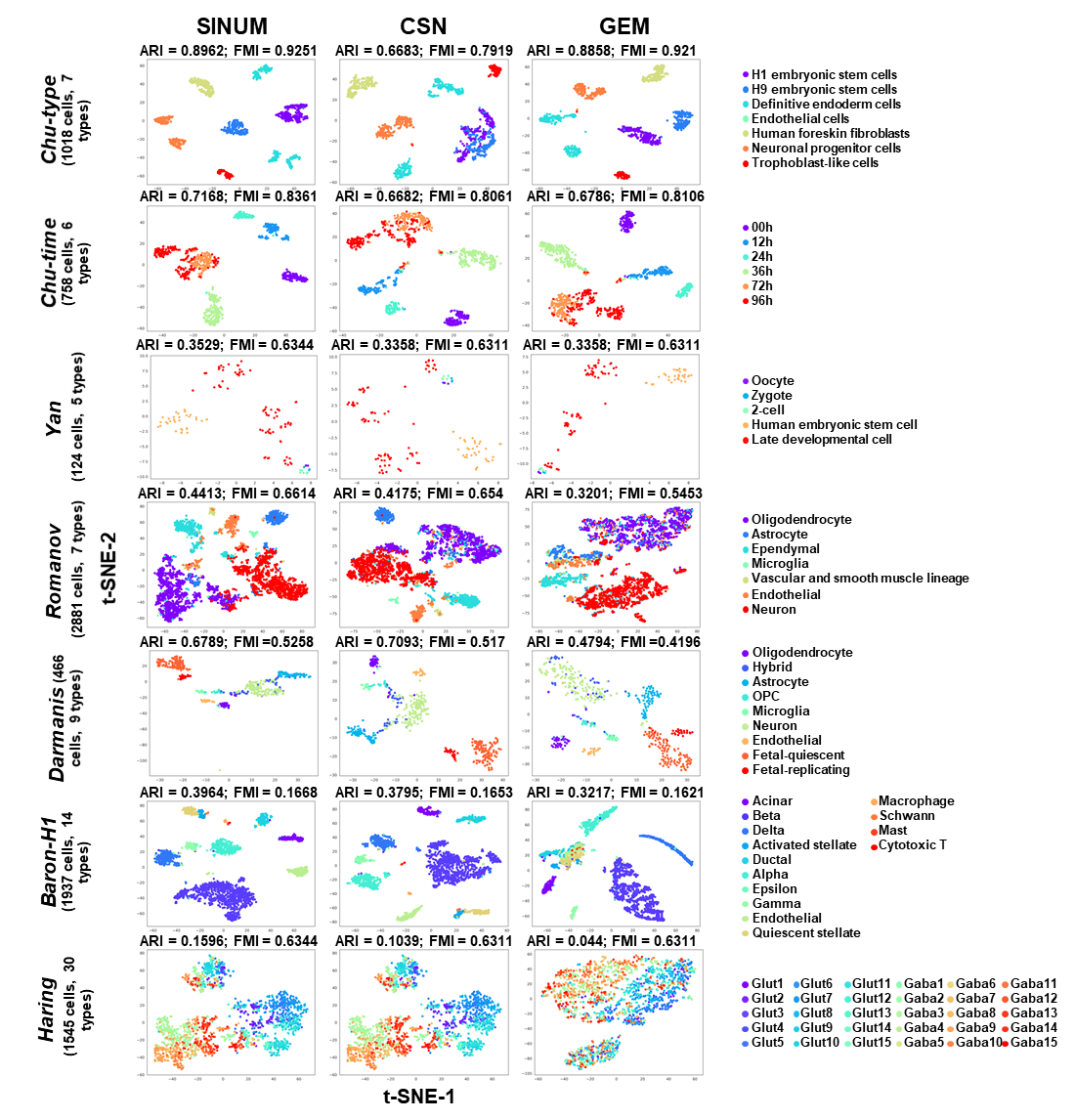


**Figure S3. t-SNE plots for visualizing the hierarchical clustering performances of SINUM DMs, CSN DMs, and GEMs on seven scRNA-seq datasets.**

For visualization, we applied t-distributed stochastic neighbor embedding (t-SNE) to reduce the dimensions to two after reducing by principal component analysis (PCA) to 20 dimensions. The *x*-axis and *y*-axis in each plot represent t-SNE-1 and t-SNE-2, respectively. For each dataset, distinct cell types are marked by different colors. Adjusted rand index (ARI) and F-measure index (FMI) were employed in comparison of clustering performances since the cell type labels of each dataset had been known.
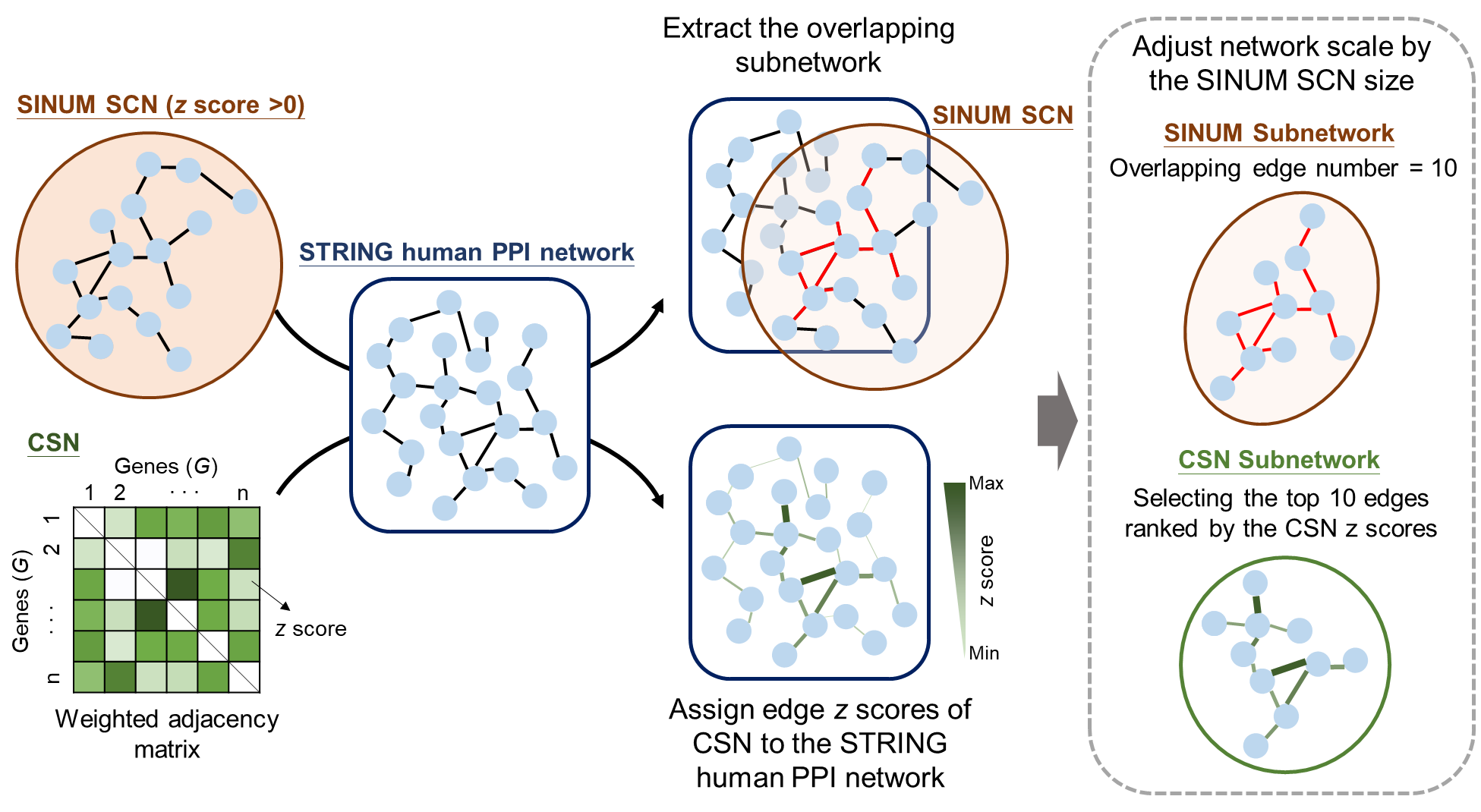


**Figure S4. The workflow of adjusting network scale by the SINUM SCN size.**

First, the overlapping subnetwork (i.e., intersection) between each SINUM SCN and the STRING human PPI network was extracted. Next, the edge z scores of CSN were assigned to all the edges of the STRING human PPI network. Finally, according to the number of edges in each overlapping subnetwork of SINUM, the same edge number from the STRING human PPI network was further selected in descending order of edge z scores of CSN to build the corresponding subnetwork of CSN.


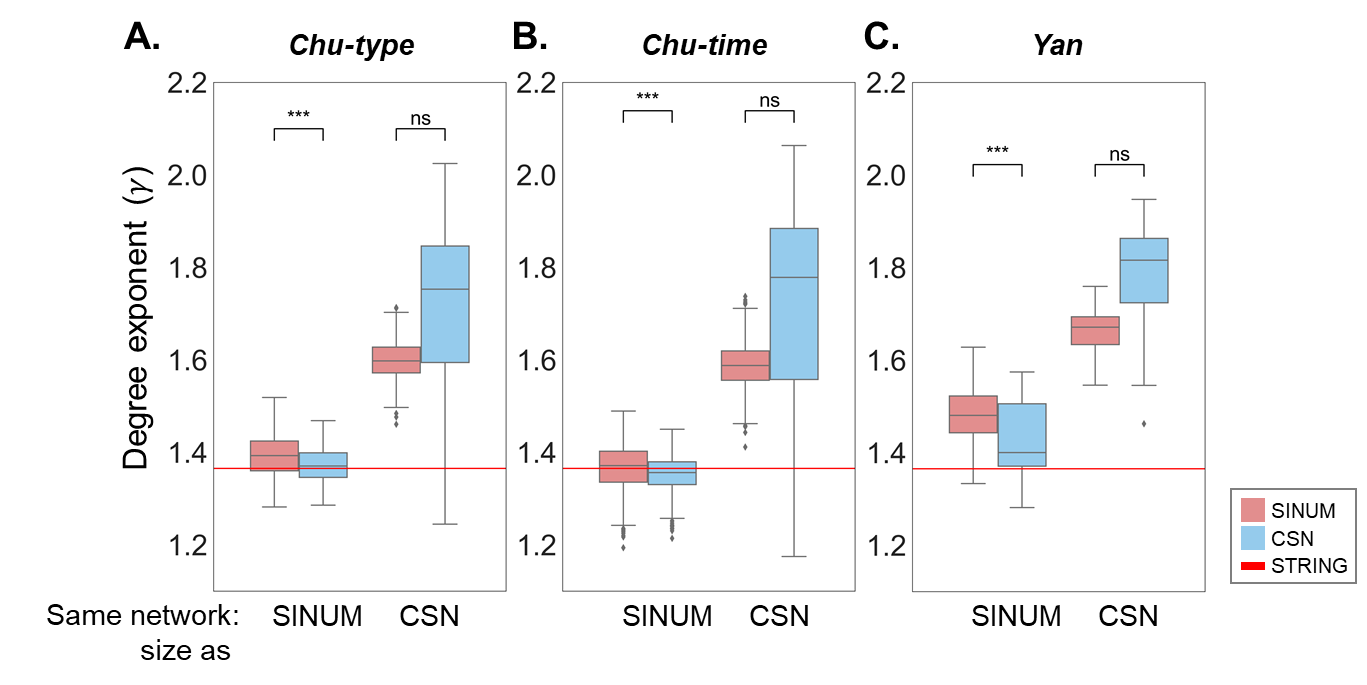


**Figure S5. Distribution of degree exponent (**$\boldsymbol{\gamma}$**) values for the SINUM (red) and CSN (blue) SCNs constructed using (A) *Chu-type*, (B) *Chu-time*, and (C) *Yan* datasets.**

The scale-free network characteristics can be described as $P\left( k \right)\sim k^{-\gamma}$, in which the probability of a node with *k* links decreases as the node degree increases on a log–log plot. To avoid network size bias, each SCN was generated by SINUM (or CSN) method via selecting the edge sorted by respective confidence scores and had the same edge number (i.e., same network size) as the original CSN (or SINUM) SCN. Next, these SCNs were intersected with the STRING human PPI network to evaluate the $\gamma$ values. The red line represents the $\gamma$ value for the STRING human PPI network.

**
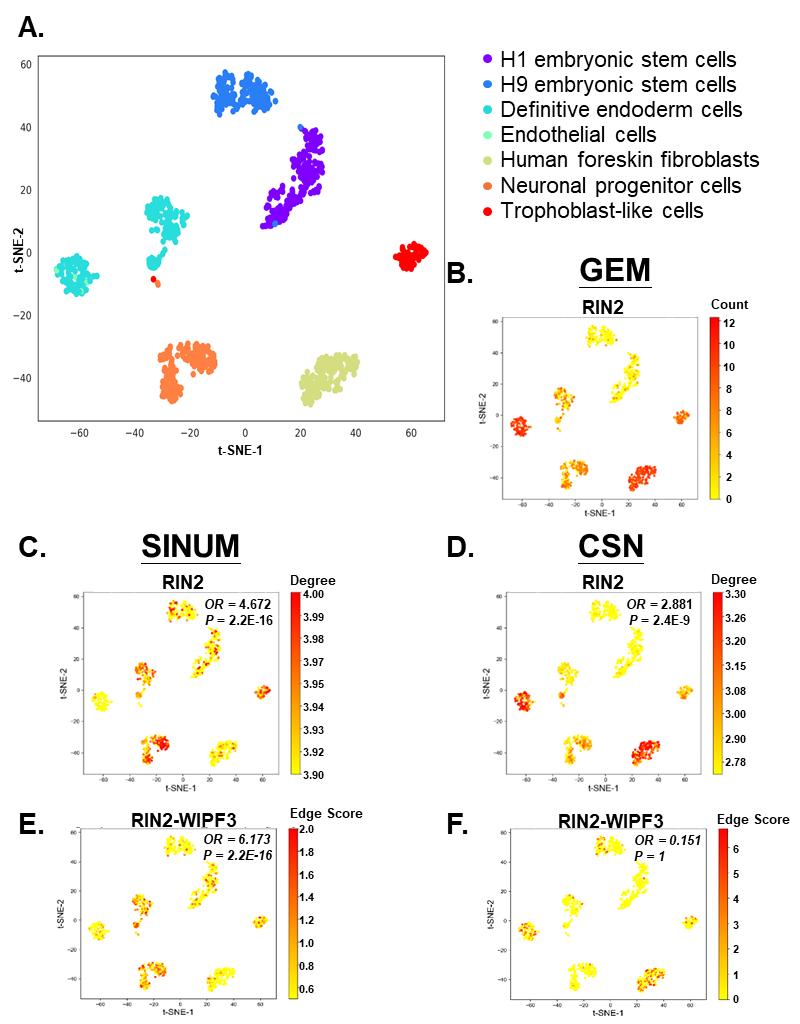
**

**Figure S6. Detection comparison of cell-type markers in neuronal progenitor cells (NPC) using the SINUM and CSN DMs and GEMs based on *Chu-Type* dataset.**

(A) t-SNE plot of GEM for *Chu-type* dataset, colored by cell types (ARI = 0.89 and FMI = 0.92). The cell type of NPC (orange) was chosen to detect the potential markers using the SINUM DMs, CSN DMs, and GEMs, such as gene *RIN2* (Ras and Rab interactor 2) and gene pair *RIN2-WIPF3*. Gene *RIN2* in the *t*-SNE plots, colored by (B) the gene expression level and the network degree level in the (C) SINUM SCNs and (D) in the SINUM SCNs. The degree *d* for networks and raw count *r* for gene expression were transformed by log_10_(*d+1*) and log_2_(*r+1*), respectively. Fisher's exact test was performed to statistically test whether the proportion of gene *RIN2* as a hub in SCNs is higher among the NPCs than among the other cells. The nodes with degrees within the top 25% of all nodes were defined as the hubs (i.e., hub genes) of each SCN. The odds ratio (*OR*) and *p* value (*P*) of statistical analysis are shown. Edge *RIN2-WIPF3* (WAS/WASL-interacting protein family member 3) in the t-SNE plots, colored by the (E) SINUM edge scores (i.e., *z* scores) and (F) CSN edge scores (i.e., *z* scores). Fisher's exact test was performed to statistically test whether the proportion of edges in SCNs is higher among the NPCs than among the other cells.


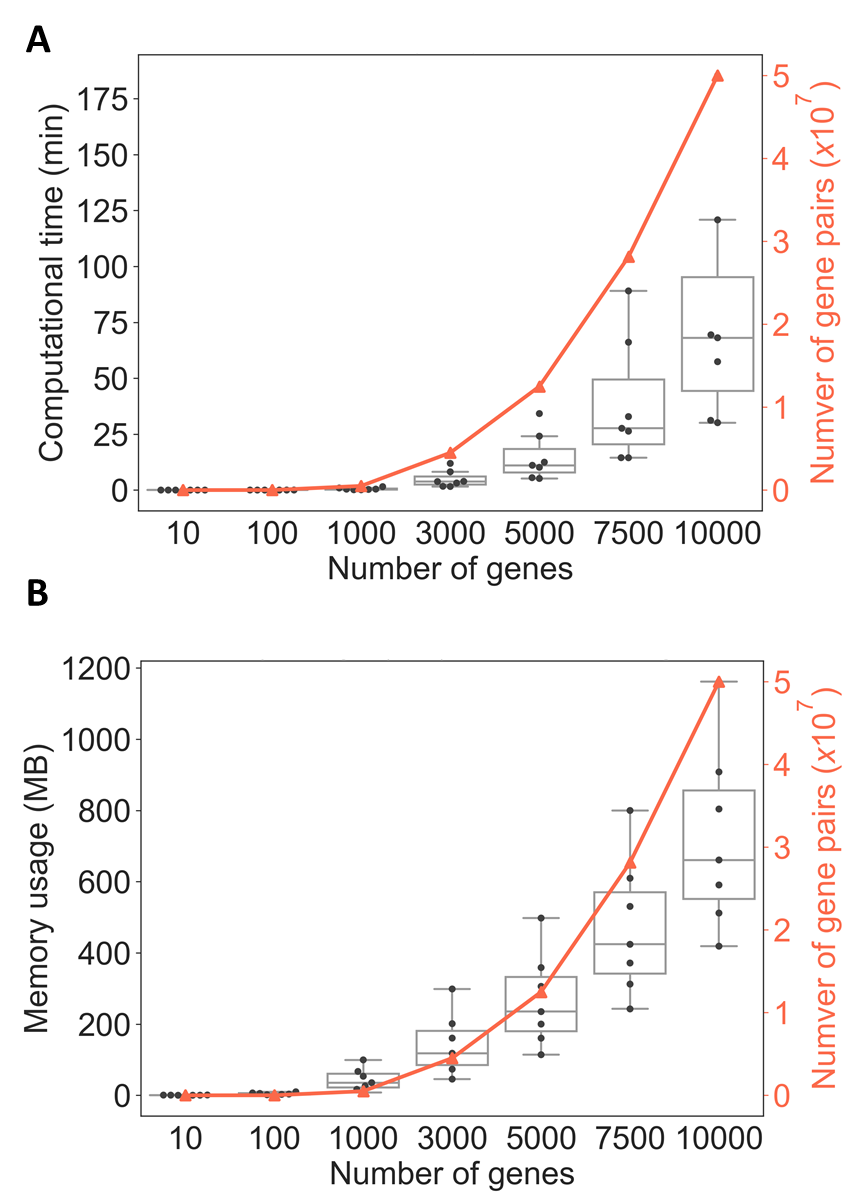


**Figure S7. (A) Computational time (min) and (B) memory usage (MB) of SINUM per cell with different numbers of genes and gene pairs on seven scRNA-seq datasets.**

In all boxplots, a data point (dot) represents the mean of computational time (or memory usage) for 30 cells randomly chosen from each scRNA-seq dataset. The computational experiments were conducted using one thread on a server with the following specifications: CentOS Linux (release 7.6.1810) operating system, with 768GB of RAM, an Intel(R) Xeon(R) Gold 5218 CPU @ 2.30GHz.

**Supplementary Tables**

**Table S1. Overlap coefficients between the edges of STRING (or Gysi) human PPI network and the SINUM (red) or CSN (blue) SCNs on seven datasets.**

| Reference PPI network | Method | Overlap coefficient (%) | | | | | | |
| --- | --- | --- | --- | --- | --- | --- | --- | --- |
|  |  | ***ChuType*** | ***ChuTime*** | ***Haring*** | ***Baron-H1*** | ***Romanov*** | ***Yan*** | ***Darmanis*** |
| STRING | SINUM | 26.39±3.61 | 24.66±4 | 6.57±3.48 | 3.97±2.3 | 6.02±5.19 | 27.09±4.67 | 10.31±6.36 |
|  | CSN | 4.4±1.34 | 3.62±1.26 | 0.88±0.75 | 0.91±0.74 | 2.31±2.76 | 8.7±3.62 | 1.44±1.16 |
| Gysi *et al.* | SINUM | 24.02±4.38 | 3.32±2.09 | 5.43±4.58 | 5.7±2.98 | 27.91±4.69 | 26.16±3.66 | 9.76±5.95 |
|  | CSN | 2.8±0.84 | 0.61±0.55 | 1.89±2.3 | 0.68±0.58 | 8.25±3.69 | 3.46±0.88 | 1.35±1.1 |

The overlap coefficient is defined as the size of the intersection divided by the smaller of the size of the two edge sets between the STRING human PPI network and each SCN. The mean ± standard deviation is shown. Because the SINUM and CSN methods were designed to detect gene-gene associations (e.g., edges) in each cell, we only evaluated the overlap coefficient between the edges of the STRING human PPI network and the networks inferred by SINUM (or CSN). Therefore, the gene (i.e., node) would be discarded in this analysis if its degree is zero. The overlap coefficients of SINUM SCNs are all significantly higher than CSN ones (*p* < 0.005, Wilcoxon signed-rank test).

**Table S2. Datasets of scRNA-seq used** **in our analysis**

| **Dataset** | **Number of cells** | **Number of cell types** | **Expression level** | **Number of genes used in this study ^a^** | **Data Sources** |
| --- | --- | --- | --- | --- | --- |
| ***Chu-type* [4]** | 1018 | 7 | count | 16619 | GSE75748 |
| ***Chu-time* [4]** | 758 | 6 |  | 15691 | GSE75748 |
| ***Haring* [5]** | 1545 | 30 |  | 14499 | GSE103840 |
| ***Baron-H1* [6]** | 1937 | 14 |  | 13274 | GSE84133 ***^b^*** (GSM2230757) |
| ***Romanov* [7]** | 2881 | 7 |  | 16112 | GSE74672 |
| ***Yan* [8]** | 124 | 5 |  | 20286 | GSE36552 |
| ***Darmanis* [9]** | 466 | 9 |  | 16818 | GSE67835 |

^a^The gene selection rule is the genes expressed in at least ten cells for each dataset.

^b^There are four human and two mouse samples in the Baron dataset. Here, we used the “Human sample 1 (GSM2230757)” for validation of SINUM.

**Table S3. Comparisons of *k*-means clustering performances for the SINUM DMs with different parameter combinations, the CSN DMs (suggested parameters, i.e., box size = 0.1 and *p* value < 0.01), and the GEM on seven scRNA-seq datasets.**

To avoid the "dropout" phenomenon in scRNA-seq data and the time-consuming construction of SCNs using the whole gene expression profiles, the FEAST algorithm was utilized to select the top 1,000 representative genes for each dataset.

| **SCN inference methods and GEM** | **Box size** | ***z* score**  **(*p* value)** | **F-measure index (the median in 30 trials)** | | | | | | |
| --- | --- | --- | --- | --- | --- | --- | --- | --- | --- |
|  |  |  | ***Haring*** | ***Baron-H1*** | ***Romanov*** | ***Darmanis*** | ***Yan*** | ***Chu-type*** | ***Chu-time*** |
| SINUM | 0.05 | -2 | 0.1938 | 0.1483 | 0.4956 | 0.2966 | 0.6333 | 0.4772 | 0.4454 |
|  | 0.05 | -1 | 0.2179 | 0.1513 | 0.5514 | 0.3562 | 0.6703 | 0.7467 | 0.5711 |
|  | 0.05 | 0 | 0.3108 | 0.1645 | 0.7389 | 0.4995 | 0.6942 | 0.8235 | 0.7926 |
|  | 0.05 | 1 | 0.2853 | 0.1640 | 0.7791 | 0.4957 | 0.6629 | 0.7764 | 0.7091 |
|  | 0.05 | 2 | 0.2376 | 0.1542 | 0.7402 | 0.4506 | 0.6788 | 0.7487 | 0.5845 |
|  | 0.1 | -2 | 0.1425 | 0.1409 | 0.4099 | 0.2254 | 0.5705 | 0.4459 | 0.3814 |
|  | 0.1 | -1 | 0.1609 | 0.1463 | 0.5034 | 0.2657 | 0.5522 | 0.5303 | 0.4502 |
|  | 0.1 | 0 | 0.3261 | 0.1637 | 0.7379 | 0.4894 | 0.7017 | 0.8944 | 0.8059 |
|  | 0.1 | 1 | 0.3171 | 0.1666 | 0.7326 | 0.4961 | 0.6942 | 0.8455 | 0.7880 |
|  | 0.1 | 2 | 0.2795 | 0.1505 | 0.7412 | 0.4761 | 0.6542 | 0.7496 | 0.4507 |
|  | 0.15 | -2 | 0.1175 | 0.1623 | 0.2736 | 0.2312 | 0.6065 | 0.4656 | 0.4282 |
|  | 0.15 | -1 | 0.1265 | 0.1451 | 0.4264 | 0.2621 | 0.5778 | 0.7012 | 0.6771 |
|  | 0.15 | 0 | 0.3220 | 0.1651 | 0.7416 | 0.4984 | 0.7017 | 0.9163 | 0.7986 |
|  | 0.15 | 1 | 0.3097 | 0.1680 | 0.7257 | 0.4850 | 0.6904 | 0.8683 | 0.7958 |
|  | 0.15 | 2 | 0.2933 | 0.1636 | 0.7492 | 0.4844 | 0.6783 | 0.7238 | 0.4187 |
|  | 0.2 | -2 | 0.1139 | 0.1556 | 0.3278 | 0.2492 | 0.6259 | 0.5170 | 0.4688 |
|  | 0.2 | -1 | 0.1323 | 0.1392 | 0.3379 | 0.3064 | 0.5549 | 0.7513 | 0.7100 |
|  | 0.2 | 0 | 0.3276 | 0.1650 | 0.7334 | 0.4963 | 0.7017 | 0.9163 | 0.8009 |
|  | 0.2 | 1 | 0.3279 | 0.1640 | 0.7191 | 0.4876 | 0.6629 | 0.8616 | 0.8009 |
|  | 0.2 | 2 | 0.3028 | 0.1627 | 0.7440 | 0.4967 | 0.6644 | 0.7200 | 0.4046 |
|  | 0.25 | -2 | 0.1090 | 0.1591 | 0.2510 | 0.2548 | 0.6508 | 0.5161 | 0.4862 |
|  | 0.25 | -1 | 0.1384 | 0.1458 | 0.2886 | 0.3253 | 0.6541 | 0.7456 | 0.7542 |
|  | 0.25 | 0 | 0.3312 | 0.1648 | 0.7314 | 0.4947 | 0.7017 | 0.9119 | 0.7927 |
|  | 0.25 | 1 | 0.3326 | 0.1662 | 0.7115 | 0.4928 | 0.6629 | 0.8510 | 0.7956 |
|  | 0.25 | 2 | 0.3189 | 0.1654 | 0.7506 | 0.4817 | 0.6665 | 0.7230 | 0.3977 |
| CSN *^a^* | 0.1 | (0.01) | 0.2623 | 0.1644 | 0.6457 | 0.5267 | 0.6311 | 0.7951 | 0.8062 |
| CSN | 0.1 | (0.01) | 0.2704 | 0.1611 | 0.7307 | 0.5004 | 0.6822 | 0.7918 | 0.8135 |
| GEM *^a^* | n/a | n/a | 0.1739 | 0.1600 | 0.5440 | 0.4355 | 0.6311 | 0.9197 | 0.8353 |
| GEM | n/a | n/a | 0.2862 | 0.1676 | 0.6878 | 0.4389 | 0.6344 | 0.9252 | 0.8010 |

*^a^* CSN DMs and the GEM include the whole genome.

**Table S4. Comparisons of hierarchical clustering performances for the SINUM DMs with different parameter combinations, the CSN DMs (suggested parameters, i.e., box size = 0.1 and *p* value < 0.01), and the GEM on seven scRNA-seq datasets.**

To avoid the "dropout" phenomenon in scRNA-seq data and the time-consuming construction of SCNs using the whole gene expression profiles, the FEAST algorithm was utilized to select the top 1,000 representative genes for each dataset.

| **SCN inference methods and GEM** | **Box size** | ***z* score**  **(*p* value)** | **F-measure index (the median in 30 trials)** | | | | | | |
| --- | --- | --- | --- | --- | --- | --- | --- | --- | --- |
|  |  |  | ***Haring*** | ***Baron-H1*** | ***Romanov*** | ***Darmanis*** | ***Yan*** | ***Chu-type*** | ***Chu-time*** |
| SINUM | 0.05 | -2 | 0.1991 | 0.1622 | 0.5009 | 0.3123 | 0.6095 | 0.4651 | 0.4349 |
|  | 0.05 | -1 | 0.2146 | 0.1597 | 0.5628 | 0.3847 | 0.6703 | 0.7508 | 0.5587 |
|  | 0.05 | 0 | 0.3040 | 0.1678 | 0.7448 | 0.5131 | 0.7091 | 0.8055 | 0.7771 |
|  | 0.05 | 1 | 0.2846 | 0.1592 | 0.7740 | 0.4983 | 0.7017 | 0.7810 | 0.6808 |
|  | 0.05 | 2 | 0.2470 | 0.1616 | 0.7134 | 0.4499 | 0.6942 | 0.7604 | 0.5396 |
|  | 0.1 | -2 | 0.1424 | 0.1553 | 0.4338 | 0.2293 | 0.5878 | 0.4549 | 0.3730 |
|  | 0.1 | -1 | 0.1619 | 0.1586 | 0.5363 | 0.2678 | 0.6347 | 0.5267 | 0.4446 |
|  | 0.1 | 0 | 0.3200 | 0.1660 | 0.7625 | 0.5159 | 0.7163 | 0.9021 | 0.8049 |
|  | 0.1 | 1 | 0.3053 | 0.1646 | 0.7560 | 0.4977 | 0.6942 | 0.8508 | 0.7900 |
|  | 0.1 | 2 | 0.2704 | 0.1654 | 0.7349 | 0.4923 | 0.6897 | 0.7544 | 0.4404 |
|  | 0.15 | -2 | 0.1160 | 0.1635 | 0.2861 | 0.2308 | 0.6047 | 0.4692 | 0.4197 |
|  | 0.15 | -1 | 0.1264 | 0.1545 | 0.4350 | 0.2566 | 0.5990 | 0.7069 | 0.6347 |
|  | 0.15 | 0 | 0.3237 | 0.1683 | 0.7576 | 0.5127 | 0.7017 | 0.9113 | 0.7939 |
|  | 0.15 | 1 | 0.3130 | 0.1681 | 0.7426 | 0.4920 | 0.6709 | 0.8443 | 0.7960 |
|  | 0.15 | 2 | 0.2902 | 0.1621 | 0.7626 | 0.5001 | 0.6703 | 0.7319 | 0.3942 |
|  | 0.2 | -2 | 0.1128 | 0.1781 | 0.3281 | 0.2500 | 0.6246 | 0.5057 | 0.4700 |
|  | 0.2 | -1 | 0.1316 | 0.1491 | 0.3306 | 0.3032 | 0.5668 | 0.7491 | 0.7073 |
|  | 0.2 | 0 | 0.3209 | 0.1689 | 0.7650 | 0.5155 | 0.7017 | 0.9133 | 0.8017 |
|  | 0.2 | 1 | 0.3201 | 0.1644 | 0.7426 | 0.4980 | 0.6629 | 0.8577 | 0.7975 |
|  | 0.2 | 2 | 0.2999 | 0.1655 | 0.7570 | 0.5129 | 0.6934 | 0.7258 | 0.3934 |
|  | 0.25 | -2 | 0.1123 | 0.2515 | 0.3096 | 0.2639 | 0.6591 | 0.5182 | 0.4818 |
|  | 0.25 | -1 | 0.1383 | 0.1469 | 0.2797 | 0.3309 | 0.6489 | 0.7502 | 0.7057 |
|  | 0.25 | 0 | 0.3212 | 0.1678 | 0.7602 | 0.5115 | 0.7017 | 0.9073 | 0.7927 |
|  | 0.25 | 1 | 0.3235 | 0.1658 | 0.7515 | 0.5031 | 0.6629 | 0.8646 | 0.7955 |
|  | 0.25 | 2 | 0.3177 | 0.1651 | 0.7653 | 0.5012 | 0.6629 | 0.7225 | 0.3813 |
| CSN *^a^* | 0.1 | (0.01) | 0.2509 | 0.1653 | 0.6541 | 0.5170 | 0.6311 | 0.7919 | 0.8062 |
| CSN | 0.1 | (0.01) | 0.2631 | 0.1658 | 0.7537 | 0.5114 | 0.6734 | 0.7822 | 0.7477 |
| GEM *^a^* | n/a | n/a | 0.1640 | 0.1621 | 0.5453 | 0.4197 | 0.6311 | 0.9210 | 0.8106 |
| GEM | n/a | n/a | 0.2918 | 0.1668 | 0.6992 | 0.4559 | 0.6344 | 0.9252 | 0.7997 |

*^a^* CSN DMs and the GEM include the whole genome.

**Table S5. Comparisons of clustering performance for the SINUM and CSN DMs and the GEM, evaluated by eight performance indexes using the median in 30 trials.**

In this analysis, the SINUM and CSN DMs and the GEM include the whole genome. The values for each performance index are the median in 30 trials due to the randomness of *t*-SNE.

| **Performance index**  **[clustering method]** | **Method** | ***Chu-type*** | ***Chu-time*** | ***Haring*** | ***Baron-H1*** | ***Romanov*** | ***Yan*** | ***Darmanis*** |
| --- | --- | --- | --- | --- | --- | --- | --- | --- |
| ***Adjusted Rand index (ARI)***  [hierarchical] | SINUM | **0.8962** | **0.716865** | **0.159632** | **0.396404** | **0.441305** | **0.352918** | 0.678907 |
|  | CSN | 0.668313 | 0.668233 | 0.103902 | 0.379558 | 0.417564 | 0.33587 | **0.709386** |
|  | GEM | 0.885806 | 0.678686 | 0.044082 | 0.321774 | 0.320114 | 0.33587 | 0.479476 |
| ***Adjusted Rand index (ARI)***  [*k*-means] | SINUM | **0.8962** | **0.730054** | **0.154658** | **0.379887** | **0.429439** | **0.414675** | 0.660336 |
|  | CSN | 0.668706 | 0.668233 | 0.106629 | 0.366148 | 0.421575 | 0.33587 | **0.73938** |
|  | GEM | 0.884532 | 0.694549 | 0.046619 | 0.319266 | 0.326747 | 0.33587 | 0.479688 |
| ***F-measure index (FMI)***  [hierarchical] | SINUM | **0.925161** | **0.836104** | **0.311988** | **0.16685** | **0.661425** | **0.634409** | **0.525828** |
|  | CSN | 0.791914 | 0.806181 | 0.250881 | 0.165327 | 0.654085 | 0.631124 | 0.517004 |
|  | GEM | 0.921002 | 0.8106 | 0.164028 | 0.162102 | 0.545346 | 0.631124 | 0.419683 |
| ***F-measure index (FMI)***  [*k*-means] | SINUM | **0.925161** | **0.858318** | **0.291413** | **0.166169** | **0.664256** | **0.658061** | 0.51647 |
|  | CSN | 0.795053 | 0.806181 | 0.262292 | 0.164415 | 0.645682 | 0.631124 | **0.526695** |
|  | GEM | 0.91965 | 0.835323 | 0.173943 | 0.160041 | 0.544041 | 0.631124 | 0.435525 |
| ***Adjusted mutual information (AMI)***  [hierarchical] | SINUM | **0.944106** | **0.852267** | **0.34422** | **0.74998** | **0.54013** | **0.553819** | 0.753317 |
|  | CSN | 0.802638 | 0.767913 | 0.26932 | 0.73118 | 0.506704 | 0.539948 | **0.788798** |
|  | GEM | 0.929614 | 0.789389 | 0.140555 | 0.65317 | 0.380143 | 0.539948 | 0.657336 |
| ***Adjusted mutual information (AMI)***  [*k*-means] | SINUM | **0.944106** | **0.851045** | **0.344162** | **0.745423** | **0.546039** | **0.580061** | 0.737432 |
|  | CSN | 0.803068 | 0.772198 | 0.272628 | 0.725348 | 0.51096 | 0.539948 | **0.795349** |
|  | GEM | 0.928398 | 0.795538 | 0.142535 | 0.652252 | 0.397262 | 0.539948 | 0.651929 |
| ***Completeness scores (CPT)***  [hierarchical] | SINUM | **0.911046** | **0.867422** | **0.397609** | **0.653752** | **0.500305** | **0.46888** | 0.752268 |
|  | CSN | 0.776198 | 0.786999 | 0.330985 | 0.634918 | 0.474802 | 0.45962 | **0.772836** |
|  | GEM | 0.897655 | 0.802019 | 0.2155 | 0.566489 | 0.350235 | 0.45962 | 0.636878 |
| ***Completeness scores (CPT)***  [*k*-means] | SINUM | **0.911046** | **0.862636** | **0.395501** | **0.646862** | **0.503445** | **0.506825** | 0.733004 |
|  | CSN | 0.775926 | 0.783662 | 0.33218 | 0.628264 | 0.4754 | 0.45962 | **0.777862** |
|  | GEM | 0.896306 | 0.804856 | 0.216596 | 0.564977 | 0.36258 | 0.45962 | 0.630001 |
| ***Fowlkes-Mallows scores (FMS)***  [hierarchical] | SINUM | **0.91605** | **0.772738** | **0.195273** | **0.540181** | **0.564066** | **0.584929** | 0.732788 |
|  | CSN | 0.725573 | 0.732781 | 0.141304 | 0.525027 | 0.545848 | 0.57245 | **0.759026** |
|  | GEM | 0.90728 | 0.741604 | 0.083122 | 0.46919 | 0.462538 | 0.57245 | 0.563143 |
| ***Fowlkes-Mallows scores (FMS)***  [*k*-means] | SINUM | **0.91605** | **0.781975** | **0.189912** | **0.526714** | **0.553956** | **0.634907** | 0.717208 |
|  | CSN | 0.725895 | 0.732781 | 0.143309 | 0.514112 | 0.548786 | 0.57245 | **0.784528** |
|  | GEM | 0.906273 | 0.752913 | 0.084912 | 0.467229 | 0.466992 | 0.57245 | 0.563586 |
| ***Homogeneity scores (HMG)***  [hierarchical] | SINUM | **0.980863** | **0.839218** | **0.412286** | **0.894478** | **0.592828** | **0.758712** | 0.77181 |
|  | CSN | 0.835048 | 0.762426 | 0.342402 | 0.87426 | 0.545966 | 0.738108 | **0.823588** |
|  | GEM | 0.965356 | 0.781168 | 0.224362 | 0.789646 | 0.422316 | 0.738108 | 0.706905 |
| ***Homogeneity scores (HMG)***  [*k*-means] | SINUM | **0.980863** | **0.844358** | **0.414577** | **0.893042** | **0.604091** | **0.74786** | 0.761029 |
|  | CSN | 0.836426 | 0.765413 | 0.34725 | 0.87313 | 0.556625 | 0.738108 | **0.830253** |
|  | GEM | 0.964419 | 0.791294 | 0.227469 | 0.791042 | 0.444629 | 0.738108 | 0.703834 |
| ***Normalized mutual information (NMI)***  [hierarchical] | SINUM | **0.944666** | **0.853723** | **0.404766** | **0.75498** | **0.541825** | **0.579581** | 0.762079 |
|  | CSN | 0.804617 | 0.770211 | 0.336595 | 0.736544 | 0.508554 | 0.566488 | **0.7968** |
|  | GEM | 0.93032 | 0.791456 | 0.219846 | 0.660078 | 0.382414 | 0.566488 | 0.669998 |
| ***Normalized mutual information (NMI)***  [*k*-means] | SINUM | **0.944666** | **0.852513** | **0.404786** | **0.750496** | **0.547705** | **0.602678** | 0.746733 |
|  | CSN | 0.805041 | 0.77443 | 0.339735 | 0.730808 | 0.512775 | 0.566488 | **0.803104** |
|  | GEM | 0.929116 | 0.79754 | 0.221858 | 0.659168 | 0.399434 | 0.566488 | 0.66473 |
| ***V-measure scores (VMS)***  *Hierarchical* | SINUM | **0.944666** | **0.853723** | **0.404766** | **0.75498** | **0.541825** | **0.579581** | 0.762079 |
|  | CSN | 0.804617 | 0.770211 | 0.336595 | 0.736544 | 0.508554 | 0.566488 | **0.7968** |
|  | GEM | 0.93032 | 0.791456 | 0.219846 | 0.660078 | 0.382414 | 0.566488 | 0.669998 |
| ***V-measure scores (VMS)***  [*k*-means] | SINUM | **0.944666** | **0.852513** | **0.404786** | **0.750496** | **0.547705** | **0.602678** | 0.746733 |
|  | CSN | 0.805041 | 0.77443 | 0.339735 | 0.730808 | 0.512775 | 0.566488 | **0.803104** |
|  | GEM | 0.929116 | 0.79754 | 0.221858 | 0.659168 | 0.399434 | 0.566488 | 0.66473 |

**Table S6. Embryo development-related genes used in this study**

| **Gene name** | **References** |
| --- | --- |
| BMP4 | [1, 4, 10, 11] |
| NODAL | [4, 10] |
| EOMES | [4] |
| MSX2 | [4] |
| CDX1 | [4, 10] |
| CER1 | [4, 10] |
| GATA4 | [4] |
| DKK4 | [4] |
| MYCT1 | [4] |
| PRDM1 | [4] |
| POU2AF1 | [4] |
| POU5F1 | [1, 4, 10-12] |
| SOX17^b^ | [4, 10] |
| CXCR4^b^ | [4, 10] |
| ID1 | [4] |
| INHBA^b^ | [4] |
| INHBE | [4] |
| NOG | [1, 11] |
| NANOG | [1, 11, 12] |
| MSX1 | [1, 11] |
| LIN28A | [1, 11] |
| ID3 | [1, 11] |
| ID2 | [1, 11] |
| GATA6 | [1, 11] |
| CDH1 | [1, 10, 11] |
| CALR | [1, 11] |
| BMPER | [1, 11] |
| VCL | [1, 11] |
| TBX3 | [1, 11] |
| SRC | [1, 11] |
| SOX7 | [1, 10, 11] |
| SOX2 | [1, 10-12] |
| SNAI2 | [1, 11] |
| PLAU | [1, 11] |
| PARP1 | [1, 11] |
| FGF8^b^ | [10] |
| WNT3^b^ | [10] |
| T | [10] |
| FGF4^a^ | [10] |
| SNAI1 | [10] |
| MIXL1 | [10] |
| LHX1 | [10] |
| GSC^b^ | [10] |
| FOXA2^b^ | [10] |
| CDH2 | [10] |
| SOX1^a^ | [10] |
| ZIC1^a^ | [10] |
| FOXF1 | [10] |
| MEOX1^b^ | [10] |
| KDR | [10] |
| CXCL12 | [10] |
| CDX2^b^ | [10] |
| CGB5^a^ | [10] |
| CASP10 | [10] |
| GUSB | [10] |
| TBP | [10] |
| KLF4^b^ | [12] |
| MYC | [12] |

^a^Note that four out of 58 genes are not expressed or expressed in a small number of cells (< 10 cells) on the *Chu-time* dataset and would be discarded in this analysis.

^b^There are 10 genes having associations with the other genes in SINUM SCNs in less than 70% of cells at the six time points in *Chu-time* dataset.

**Table S7. Comparisons of clustering performance for the SINUM and CSN DMs constructed by only estimating the edges that existed in the reference network (STRING), evaluated by eight performance indexes using the median in 30 trials.**

In this analysis, the SINUM and CSN SCNs and DMs include only the edges that existed in the STRING human PPI network. The values for each performance index are the median in 30 trials due to the randomness of *t*-SNE.

| **Performance index**  **[clustering method]** | **Method** | ***Chu-type*** | ***Chu-time*** | ***Haring*** | ***Baron-H1*** | ***Romanov*** | ***Yan*** | ***Darmanis*** | ***10xPBMC^a^*** |
| --- | --- | --- | --- | --- | --- | --- | --- | --- | --- |
| ***Adjusted Rand index (ARI)***  [hierarchical] | SINUM | **0.669985** | **0.446569** | 0.022216 | **0.077979** | **0.325514** | **0.340627** | **0.526979** | **0.199417** |
|  | CSN | 0.223579 | 0.131674 | **0.0268** | 0.05084 | 0.148356 | 0.323449 | 0.389746 | 0.160977 |
| ***Adjusted Rand index (ARI)***  [*k*-means] | SINUM | **0.667973** | **0.444969** | 0.022042 | **0.082465** | **0.293636** | 0.318246 | **0.504449** | **0.2147** |
|  | CSN | 0.209348 | 0.120726 | **0.026281** | 0.045627 | 0.140195 | **0.33222** | 0.381006 | 0.155691 |
| ***F-measure index (FMI)***  [hierarchical] | SINUM | **0.764307** | **0.516278** | 0.024828 | **0.093887** | **0.373815** | **0.399402** | **0.614292** | **0.231432** |
|  | CSN | 0.25758 | 0.156184 | **0.03076** | 0.06309 | 0.172048 | 0.374848 | 0.448912 | 0.186253 |
| ***F-measure index (FMI)***  [*k*-means] | SINUM | **0.768934** | **0.512162** | 0.024665 | **0.096001** | **0.335217** | 0.363196 | **0.58434** | **0.244791** |
|  | CSN | 0.242826 | 0.136701 | **0.031838** | 0.054777 | 0.177801 | **0.398731** | 0.444579 | 0.179697 |
| ***Adjusted mutual information (AMI)***  [hierarchical] | SINUM | **0.807437** | **0.557695** | 0.08208 | **0.242618** | **0.352171** | **0.511752** | **0.646573** | **0.391899** |
|  | CSN | 0.368333 | 0.193784 | **0.091211** | 0.157684 | 0.20904 | 0.493322 | 0.513328 | 0.330935 |
| ***Adjusted mutual information (AMI)***  [*k*-means] | SINUM | **0.801919** | **0.544604** | 0.084308 | **0.24926** | **0.332136** | 0.489231 | **0.621749** | **0.398509** |
|  | CSN | 0.33863 | 0.178589 | **0.092191** | 0.150705 | 0.197013 | **0.509909** | 0.523053 | 0.322695 |
| ***Completeness scores (CPT)***  [hierarchical] | SINUM | **0.782698** | **0.554657** | 0.163627 | **0.22037** | **0.326818** | **0.438967** | **0.635408** | **0.40019** |
|  | CSN | 0.368626 | 0.204335 | **0.171706** | 0.149857 | 0.194089 | 0.425863 | 0.503742 | 0.337654 |
| ***Completeness scores (CPT)***  [*k*-means] | SINUM | **0.777511** | **0.540003** | 0.165105 | **0.225259** | **0.304887** | 0.419015 | **0.609671** | **0.401698** |
|  | CSN | 0.336051 | 0.185919 | **0.171935** | 0.142807 | 0.181521 | **0.435299** | 0.509818 | 0.326435 |
| ***Fowlkes-Mallows scores (FMS)***  [hierarchical] | SINUM | **0.72699** | **0.545387** | 0.062205 | **0.218862** | **0.469219** | **0.576369** | **0.604282** | **0.278749** |
|  | CSN | 0.360576 | 0.296532 | **0.066871** | 0.192314 | 0.319477 | 0.564497 | 0.484719 | 0.24385 |
| ***Fowlkes-Mallows scores (FMS)***  [*k*-means] | SINUM | **0.725418** | **0.543129** | 0.060979 | **0.221094** | **0.44011** | 0.557494 | **0.584564** | **0.289413** |
|  | CSN | 0.345662 | 0.280824 | **0.065078** | 0.181615 | 0.3094 | **0.568853** | 0.476966 | 0.236723 |
| ***Homogeneity scores (HMG)***  [hierarchical] | SINUM | **0.838668** | **0.567812** | 0.170517 | **0.309638** | **0.388292** | **0.705322** | **0.684383** | **0.387233** |
|  | CSN | 0.383262 | 0.19998 | **0.178806** | 0.208605 | 0.23221 | 0.683962 | 0.561946 | 0.327321 |
| ***Homogeneity scores (HMG)***  [*k*-means] | SINUM | **0.832179** | **0.558151** | 0.173109 | **0.319044** | **0.370623** | 0.680654 | **0.6643** | **0.39787** |
|  | CSN | 0.354983 | 0.187118 | **0.180678** | 0.202612 | 0.222444 | **0.704825** | 0.575061 | 0.322415 |
| ***Normalized mutual information (NMI)***  [hierarchical] | SINUM | **0.809374** | **0.56191** | 0.166907 | **0.25767** | **0.35455** | **0.539853** | **0.659822** | **0.393456** |
|  | CSN | 0.374763 | 0.201646 | **0.175217** | 0.174462 | 0.211936 | 0.522574 | 0.531255 | 0.33265 |
| ***Normalized mutual information (NMI)***  [*k*-means] | SINUM | **0.803911** | **0.548927** | 0.169009 | **0.264144** | **0.334557** | 0.518709 | **0.635865** | **0.400035** |
|  | CSN | 0.34531 | 0.186517 | **0.176198** | 0.167532 | 0.19991 | **0.538204** | 0.540486 | 0.324412 |
| ***V-measure scores (VMS)***  *Hierarchical* | SINUM | **0.809374** | **0.56191** | 0.166907 | **0.25767** | **0.35455** | **0.539853** | **0.659822** | **0.393456** |
|  | CSN | 0.374763 | 0.201646 | **0.175217** | 0.174462 | 0.211936 | 0.522574 | 0.531255 | 0.33265 |
| ***V-measure scores (VMS)***  [*k*-means] | SINUM | **0.803911** | **0.548927** | 0.169009 | **0.264144** | **0.334557** | 0.518709 | **0.635865** | **0.400035** |
|  | CSN | 0.34531 | 0.186517 | **0.176198** | 0.167532 | 0.19991 | **0.538204** | 0.540486 | 0.324412 |

**^a^** *10x PBMC dataset* (<https://support.10xgenomics.com/single-cell-gene-expression/datasets>), including 13 cell types and 11,996 cells. The counts of 18,873 genes were used as the input.

**References**

1. Dai H, Li L, Zeng T, Chen L: Cell-specific network constructed by single-cell RNA sequencing data. *Nucleic Acids Research* 2019, 47(11):e62-e62.

2. Li L, Dai H, Fang Z, Chen L: c-CSN: Single-cell RNA Sequencing Data Analysis by Conditional Cell-specific Network. *Genomics, Proteomics & Bioinformatics* 2021, 19(2):319-329.

3. Dai H, Li L, Zeng T, Chen LN: Cell-specific network constructed by single-cell RNA sequencing data. *Nucleic Acids Res* 2019, 47(11).

4. Chu L-F, Leng N, Zhang J, Hou Z, Mamott D, Vereide DT, Choi J, Kendziorski C, Stewart R, Thomson JA: Single-cell RNA-seq reveals novel regulators of human embryonic stem cell differentiation to definitive endoderm. *Genome Biology* 2016, 17(1):173.

5. Häring M, Zeisel A, Hochgerner H, Rinwa P, Jakobsson JET, Lönnerberg P, La Manno G, Sharma N, Borgius L, Kiehn O *et al*: Neuronal atlas of the dorsal horn defines its architecture and links sensory input to transcriptional cell types. *Nature Neuroscience* 2018, 21(6):869-880.

6. Baron M, Veres A, Wolock Samuel L, Faust Aubrey L, Gaujoux R, Vetere A, Ryu Jennifer H, Wagner Bridget K, Shen-Orr Shai S, Klein Allon M *et al*: A Single-Cell Transcriptomic Map of the Human and Mouse Pancreas Reveals Inter- and Intra-cell Population Structure. *Cell Systems* 2016, 3(4):346-360.e344.

7. Romanov RA, Zeisel A, Bakker J, Girach F, Hellysaz A, Tomer R, Alpár A, Mulder J, Clotman F, Keimpema E *et al*: Molecular interrogation of hypothalamic organization reveals distinct dopamine neuronal subtypes. *Nature Neuroscience* 2017, 20(2):176-188.

8. Yan L, Yang M, Guo H, Yang L, Wu J, Li R, Liu P, Lian Y, Zheng X, Yan J *et al*: Single-cell RNA-Seq profiling of human preimplantation embryos and embryonic stem cells. *Nature Structural & Molecular Biology* 2013, 20(9):1131-1139.

9. Darmanis S, Sloan SA, Zhang Y, Enge M, Caneda C, Shuer LM, Hayden Gephart MG, Barres BA, Quake SR: A survey of human brain transcriptome diversity at the single cell level. *Proceedings of the National Academy of Sciences* 2015, 112(23):7285-7290.

10. D'Amour KA, Agulnick AD, Eliazer S, Kelly OG, Kroon E, Baetge EE: Efficient differentiation of human embryonic stem cells to definitive endoderm. *Nature Biotechnology* 2005, 23(12):1534-1541.

11. Guo W-F, Yu X, Shi Q-Q, Liang J, Zhang S-W, Zeng T: Performance assessment of sample-specific network control methods for bulk and single-cell biological data analysis. *PLoS computational biology* 2021, 17(5):e1008962.

12. Zhao W, Ji X, Zhang F, Li L, Ma L: Embryonic Stem Cell Markers. In: *Molecules.* vol. 17; 2012: 6196-6236.
